# UHPLC–Q–Orbitrap–HRMS-Based Multilayer Mapping of the Pharmacodynamic Substance Basis and Mechanistic Landscape of Maizibizi Wan in Chronic Nonbacterial Prostatitis Therapy

**DOI:** 10.3390/ph19010153

**Published:** 2026-01-15

**Authors:** Maimaitiming Maihemuti, Muaitaer Nuermaimaiti, Wuermaitihan Maimaitiming, Alimujiang Paierhati, Hailong Ji, Muhammatjan Abduwaki, Xinzhou Yang, Nabijan Mohammadtursun

**Affiliations:** 1 Xinjiang Key Laboratory of Hetian Characteristic Chinese Traditional Medicine Research, Xinjiang Hetian College, Hetian 848000, China; mnmahmut@163.com (M.M.); muaitaer@sina.com (M.N.); wormatgul118@163.com (W.M.); muhammatjan76@163.com (M.A.); 2 School of Pharmacy, Xinjiang Hetian College, Hetian 848000, China; 3 School of Pharmacy, South-Central University for Nationalities, Wuhan 430074, China; xzyang@mail.scuec.edu.cn

**Keywords:** Maizibiziwan, chronic non-bacterial prostatitis, UHPLC-Q-Orbitrap-HRMS, blood-absorbable components, GNPS, network pharmacology, molecular docking

## Abstract

**Background:** Chronic nonbacterial prostatitis (CNP), the major subset of chronic prostatitis/chronic pelvic pain syndrome (CP/CPPS), imposes a substantial global burden yet lacks satisfactory therapies. Maizibizi Wan (MZBZ) has long been used clinically for prostatitis, but its pharmacodynamic substance basis and mechanisms remain unclear. **Methods**: Ultra-high-performance liquid chromatography–Q-Orbitrap high-resolution mass spectrometry (UHPLC-Q-Orbitrap-HRMS) coupled with Global Natural Products Social Molecular Networking (GNPS) molecular networking profiled MZBZ constituents and rat plasma–exposed prototype components and metabolites was used. Based on blood-absorbable components, network pharmacology predicted core targets/pathways; representative interactions were validated by molecular docking. A λ-carrageenan–induced CNBP rat model underwent histopathology (H&E), serum cytokine assays (TNF-α, IL-1β, IL-6/IL-17), immunohistochemistry (COX-2, TNF-α, MMP-9), and Western blotting (P-p65/p65, p-AKT/AKT, COX-2, TGF-β1, BCL2). **Results**: A total of 188 chemical constituents were identified in MZBZ (79 flavonoids, 38 organic acids, 30 alkaloids, 15 phenylpropanoids, 7 steroids, 4 phenylethanoid glycosides, 15 others). A total of 35 blood-absorbable components (18 prototype components, 17 metabolites) were identified, mainly involving Phase I oxidation and Phase II glucuronidation/sulfation. Network analysis yielded 54 core targets enriched in NF-κB and PI3K/AKT signaling and apoptosis. Docking indicated stable binding of key flavonoids to COX-2, NFKB1, TNF, IL-6, and BCL2. In vivo, MZBZ ameliorated prostatic inflammation, reduced serum TNF-α/IL-1β/IL-6/IL-17 (*p* < 0.05 or *p* < 0.01); decreased P-p65/p65, p-AKT/AKT, COX-2, and TGF-β1; and increased BCL2 in prostate tissue. **Conclusions**: MZBZ exerts anti-CNBP effects via multi-component synergy (prototypes + metabolites) that suppresses inflammatory cytokines, modulates apoptosis, and inhibits NF-κB and PI3K/AKT pathways. These findings provide a mechanistic basis and quality control cues for the rational clinical use of MZBZ.

## 1. Introduction

Chronic prostatitis/chronic pelvic pain syndrome (CP/CPPS) is a common yet poorly understood urological disorder that imposes a substantial global health burden. Characterized by pelvic pain, lower urinary tract symptoms, and sexual dysfunction, CP/CPPS significantly impairs quality of life and is associated with increased healthcare utilization and psychological comorbidities such as anxiety and depression [1,2]. Although not life-threatening, its chronicity and symptom complexity make it a major contributor to male morbidity worldwide. Epidemiological studies indicate that CP/CPPS affects approximately 8.2% of men globally, with prevalence rates ranging from 2.2% to 9.7% across different populations [3,4]. A 2025 meta-analysis of 26 Chinese surveys (20,127 men) reported that 59% of CP/CPPS patients suffer sexual dysfunction—34% erectile dysfunction (ED) and 35% premature ejaculation—highlighting a heavy national disease burden [5]. Nationwide multicity studies showed the community prevalence of prostatitis-like symptoms was 8.4% (12,743 participants), with 4.5% meeting criteria for chronic nonbacterial prostatitis (CNP); peak prevalence occurred at 30–50 years and was closely linked to modifiable factors such as smoking, alcohol, and psychological stress [6]. Southern China cohorts revealed an even higher symptom rate of 12.4% (2790 men) and a strong association with ED (age-adjusted OR = 1.86, *p* < 0.0001), confirming that sexual comorbidity increases with symptom severity [7]. Overall, these data indicate chronic prostatitis is common among Chinese males, but longitudinal, nationally representative studies are still needed to clarify incidence and long-term outcomes. Notably, CP/CPPS is not just a regional issue; population-based surveys in the United States, Finland, and Canada have reported similar prevalence rates [8], emphasizing its global relevance.

According to the National Institutes of Health (NIH) classification, more than 90% of chronic prostatitis cases are nonbacterial, accounting for roughly one-quarter of all urologic outpatient visits worldwide [9]. Although triggers range from occult infection and autoimmunity to endocrine disturbance, neuroplastic changes, and psychosocial stress, mounting evidence points to sustained inflammation as the pivotal driver of disease progression [10,11].

Despite its high prevalence, current treatment options remain limited and often unsatisfactory. Conventional therapies include alpha-blockers, antibiotics, anti-inflammatory drugs, and phytotherapy, but these primarily target symptom relief rather than disease modification. For instance, antibiotics are often prescribed empirically even in the absence of bacterial infection, leading to antibiotic resistance and microbiome dysbiosis [12,13]. Alpha-blockers provide modest benefits in symptom scores but are associated with dizziness, fatigue, and ejaculatory dysfunction. Moreover, psychological therapies and neuromodulators, while helpful in some cases, are not universally effective and often require long-term commitment with variable patient adherence.

In contrast, Traditional Chinese Medicine (TCM) offers a holistic, multi-target, and multi-pathway approach that aligns well with the complex pathophysiology of CP. TCMformulations act through network pharmacology mechanisms, offering a promising alternative or adjunctive strategy for chronic, multifactorial diseases like CP. Given the limited efficacy and side effects of conventional therapies, as well as the growing evidence supporting TCM’s multi-modal therapeutic potential, there is an urgent need to elucidate the pharmacodynamic basis and molecular mechanisms of traditional formulations.

The Maizibizi Wan (Maizibizi refers to prostate in the Uyghur language, MZBZ) has undergone decades of clinical validation and has proven effective in preventing and treating prostatitis. The principal components of Maizibizi Wan include Suanjiang (calyx of franchet groundcherry, *Physalis alkekengi* L. var. *franchetii*), Longkuiguo (Fruit of Black Nightshade Herb; *Solanum nigrum* L.), Cheqianzi (Psyllium; *Plantago ovata Forssk*), Huluzi (seed of bottle gourd; *Lagenaria siceraria*), Huangguazi (seed of cucumber; *Cucumis sativus* L.), and Baqia (smilax rhizome; *Smilax china* L.). Folk experience also shows that MZBZ can relieve core symptoms such as pelvic pain and urinary difficulties caused by prostatitis in men, further highlighting its promising applications in the field of anti-prostatitis therapy.

Our previous studies revealed that MZBZ modulates key pathways involving autoimmune regulation, inflammatory response, and oxidative stress, demonstrating significant therapeutic efficacy in nonbacterial prostatitis animal models [14,15]. However, the precise bioactive constituents and pharmacokinetic profile of this polyherbal preparation remain uncharacterized, limiting its clinical translation. Here, we couple untargeted UHPLC-Q-Orbitrap-HRMS profiling with targeted plasma metabolite mining to show that their blood-accessible prototypes plus their Phase-I/II metabolites—not individual markers—are responsible for efficacy. Unlike previous reports that merely correlated chemical fingerprints with activity, we experimentally validate the network-predicted targets and pathways in a carrageenan-induced CNP model and nominate specific flavonoid metabolites as quality-control surrogates. This prototype–metabolite synergy concept represents a clear advance over single-compound-centric explanations of herbal anti-prostatitis effects.

## 2. Results

### 2.1. Chemical Profiling of MZBZ Based on LC-MS

**Figure 1:**
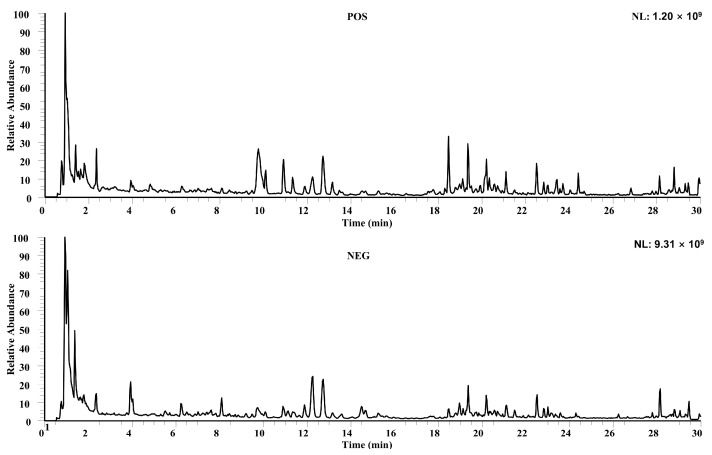
Total ion currents (TICs) of MZBZ in positive and negative ion modes.

TICs acquired in ESI± modes revealed extensive chemical diversity (Figure 1). In total, 188 constituents were annotated: 79 flavonoids (apigenin, luteolin, kaempferol, quercetin backbones), 38 organic acids (caffeoylquinic and ferulic derivatives), 30 alkaloids, 15 phenylpropanoids, 7 steroids (steroidal saponins), 4 phenylethanoid glycosides, and 15 others (Table 1).

#### 2.1.1. Flavonoids

**Figure 2:**
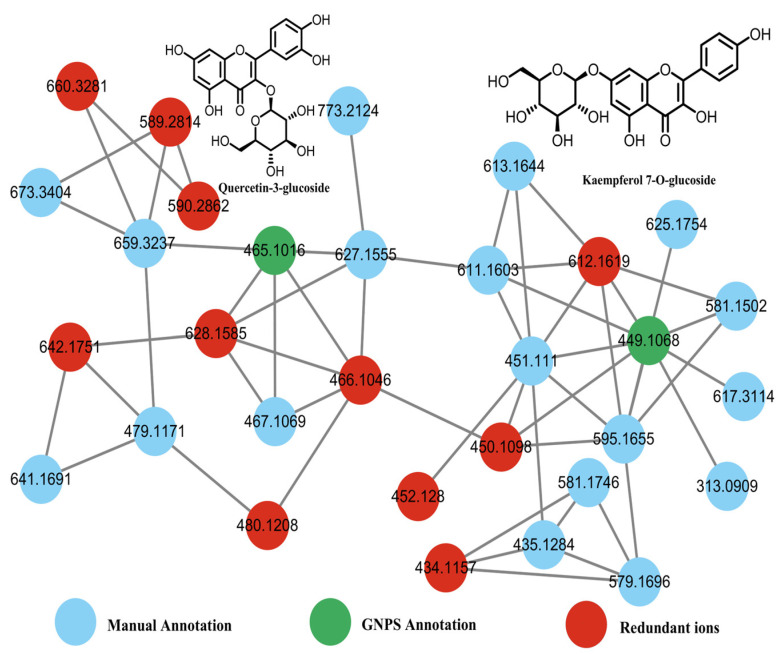
Feature-based molecular network (FBMN) of flavonoids generated on the GNPS platform.

**Figure 3:**
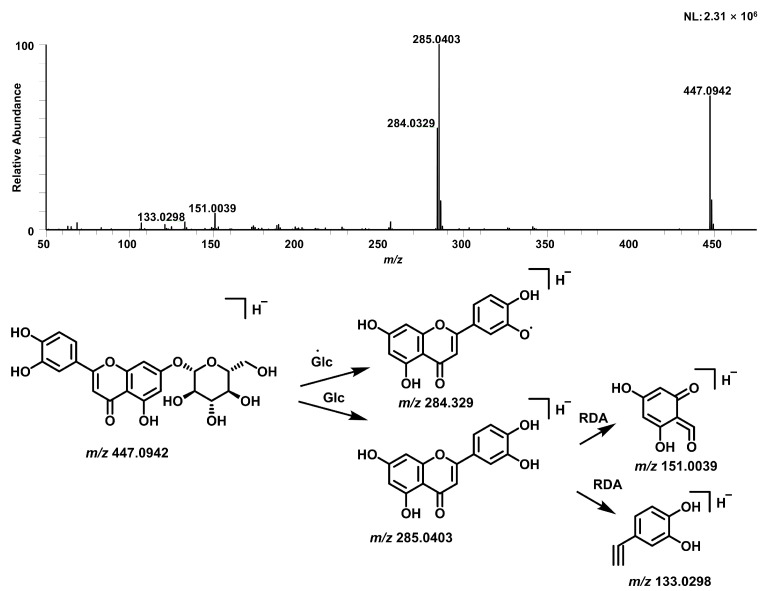
Proposed fragmentation of luteolin-7-O-glucoside (Cynaroside).

Seventy-nine flavonoid compounds were identified, primarily as glycosides of apigenin, luteolin, kaempferol, and quercetin. These compounds exhibited characteristic neutral losses of hexose (162 Da), deoxyhexose (146 Da), and pentose (132 Da). GNPS molecular networking of flavonoids revealed 33 nodes, with 2 annotated by GNPS and 20 manually annotated in Figure 2. For instance, compound 72 (*t*_R_ = 10.59 min) displayed a [M−H]^−^ ion at *m*/*z* 447.0942 (C_21_H_20_O_11_) and fragment ions at *m*/*z* 285, 284, 151, and 133. The loss of a hexose moiety (162 Da) yielded the aglycone ion at *m*/*z* 285, which was identified as luteolin based on Retro-Diels-Alder (RDA) fragmentation. Comparison with a reference standard confirmed this compound as luteolin-7-O-glucoside (Figure 3).

#### 2.1.2. Organic Acids

Thirty-eight organic acid compounds were identified, primarily caffeoylquinic acid and ferulic acid derivatives. Compounds 18, 20, 22, and 30 exhibited [M−H]^−^ ions at *m*/*z* 515.14 (C_22_H_28_O_14_) and characteristic fragment ions at *m*/*z* 353, 191, 179, and 135, indicating caffeoylquinic acid–hexoside structures. Compound 109 (*m*/*z* 515.1204, C_25_H_24_O_12_) showed similar fragments and was identified as 4,5-dicaffeoylquinic acid by comparison with a reference standard.

#### 2.1.3. Alkaloids

Thirty alkaloids were identified, mainly phenylpropamide steroidal alkaloids. Compounds 69 and 97 (*t*_R_ = 10.55 and 12.54 min) exhibited [M−H]^−^ ions at *m*/*z* 328 (C_18_H_19_NO_5_) and fragment ions at *m*/*z* 310, 295, 161, and 133, consistent with feruloyloctopamine isomers [13].

#### 2.1.4. Other Compounds

**Figure 4:**
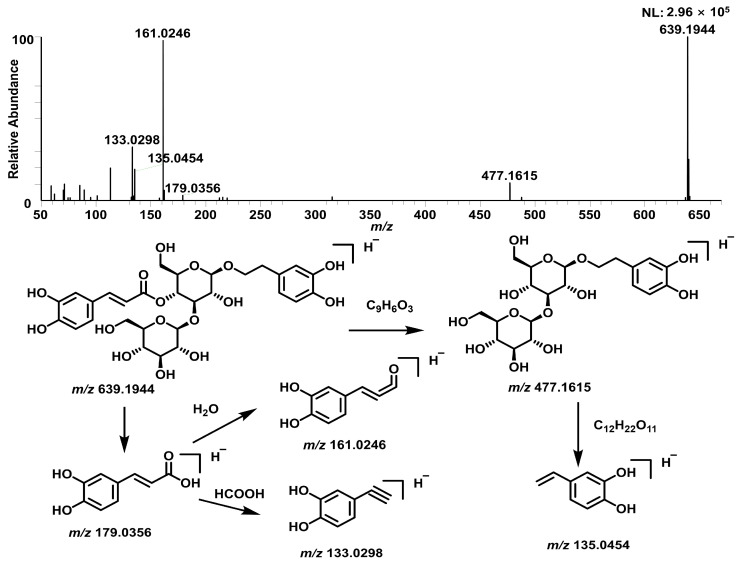
Proposed fragmentation of plantamajoside.

A diverse array of other compounds was identified, comprising fifteen phenylpropanoids, seven steroids, four phenylethanoid glycosides, and fifteen unclassified compounds. The phenylpropanoids are characterized by a core structure of cinnamic or coumaric acid esterified with sugars. For instance, compound 160 (*t*_R_ = 21.76 min, *m*/*z* 777.2268, C_36_H_42_O_19_) was preliminarily identified as Smilaside A [14]. The steroids primarily exist as steroidal saponins, exemplified by compound 151 (*t*_R_ = 20.70 min, *m*/*z* 919.4923, C_45_H_76_O_19_), which was tentatively identified as Trigoneoside Xb [15]. Furthermore, compounds 77 and 103 (both *m*/*z* 639, C_29_H_36_O_16_) were characterized as isomers of plantamajoside based on their fragmentation patterns [16]. The proposed fragmentation pathway for this compound is shown in Figure 4.

### 2.2. Plasma-Exposed Prototypes and Metabolites

**Figure 5:**
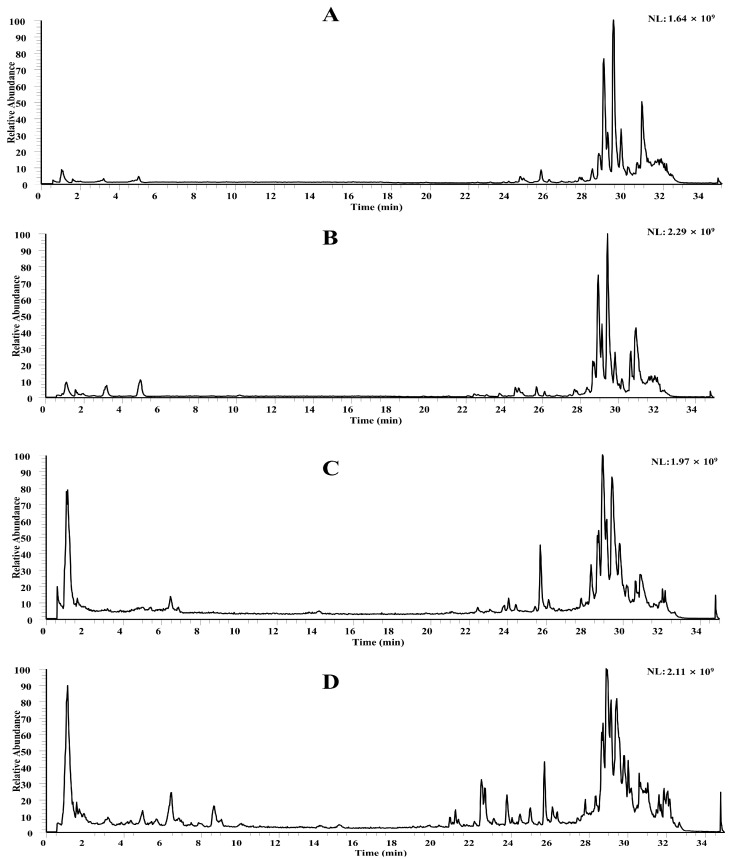
TICs of blank vs. drug-treated plasma. (**A**) blank plasma in positive ion mode, (**B**) drug-administered plasma in positive ion mode; (**C**) blank plasma in negative ion mode, (**D**) drug-administered plasma in negative ion mode.

A total of 35 plasma-exposed molecules (18 prototypes; 17 metabolites) were identified (Figure 5; Table 2). Phase II glucuronides included taxifolin-O-glucuronides ([M−H]^−^ *m*/*z* 479 → 303/285/125). Sulfated isorhamnetin metabolites ([M−H]^−^ *m*/*z* 395; C_16_H_12_O_10_S) showed neutral SO_3_ loss to *m*/*z* 315 with fragments at *m*/*z* 300/151. Putative oxidized–glucuronidated hesperetin ([M−H]^−^ *m*/*z* 493 → 317/289) was also detected. These transformations increase polarity and likely facilitate exposure to prostatic tissue.

### 2.3. Network Pharmacology Highlights

#### 2.3.1. Prediction of Targets for Blood-Absorbable Prototype Components of MZBZ

**Figure 6:**
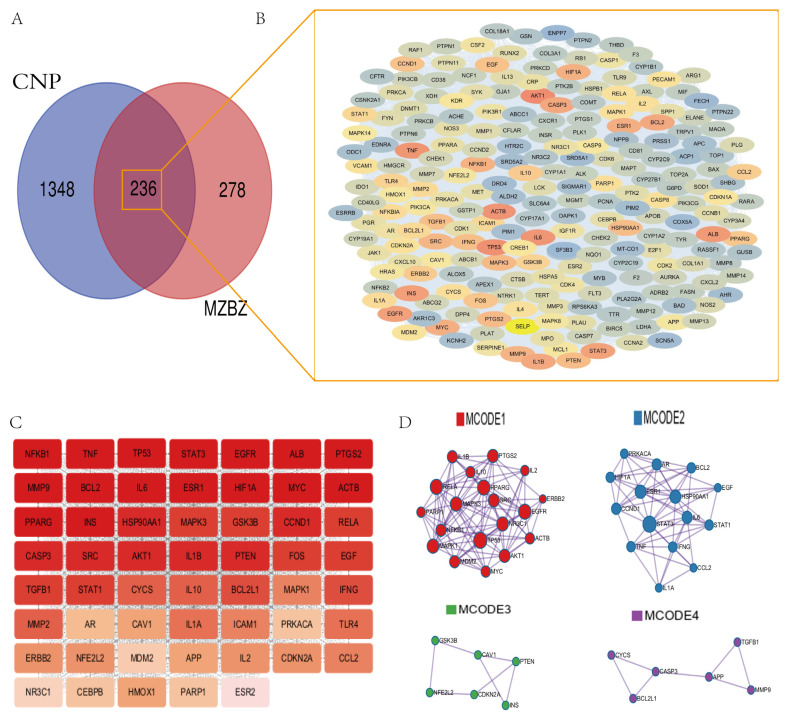
(**A**) Venn diagram of intersecting targets; (**B**) PPI network of intersected targets; (**C**) core-target PPI; (**D**) PPI modules (MCODE).

A total of 514 targets related to the 18 blood-absorbable prototype components of MZBZ were mined using databases such as Swiss Target Prediction, DrugBank, and ETCM. Furthermore, 1584 targets associated with CNBP were collected from the GeneCards, OMIM, and DrugBank databases. The MZBZ blood-absorbable component targets and CNBP targets were imported into the VENNY platform, resulting in 236 common targets. A Venn diagram was constructed, as shown in Figure 6A. To delineate the molecular cooperation among these shared targets, a protein–protein interaction (PPI) network was constructed via the STRING database (medium confidence = 0.4). After excluding two singleton nodes, the resultant network comprised 234 nodes and 7604 edges (average node degree = 64.9; clustering coefficient = 0.65; PPI enrichment *p* < 1 × 10^−16^), and was visualized in Cytoscape 3.10.3 software (Figure 6B), In Figure 6, blue to orange-red gradient to encode the topological importance of each node.

The above network was analyzed using the CentiScaPe plugin. The average values of Degree Centrality (DC), Closeness Centrality (CC), and Betweenness Centrality (BC) within the network were calculated. Targets exceeding the average values of all three parameters were sequentially screened (DC > 64.9; CC > 0.0024; BC > 174.9), identifying 54 core targets for MZBZ’s intervention in CNP, including NFKB, IL-6, PTGS2, IL-1B, TNF, and AKT1, as shown in Figure 6C. Darker red colors indicate a higher ranking in the topological analysis. Simultaneously, a Protein–Protein Interaction (PPI) analysis was performed on the core target gene co-expression network, screening for four distinct gene co-expression networks (MCODEs) using Metascape, as shown in Figure 6D. MCODE1 (red network) contains genes such as IL1B, IL10, IL2, and RELA. These genes are primarily involved in immune response and inflammatory processes, such as cytokine signaling and the NF-κB signaling pathway. MCODE2 (blue network) contains genes such as PRKACA, AR, BCL2, and EGF. These genes are associated with cell proliferation, differentiation, and survival, involving signaling pathways such as the EGFR pathway. MCODE3 (green network) contains GSK3B, CAV1, PTEN, NFE2L2, etc. These genes are related to cell cycle regulation, metabolic processes, and oxidative stress response. MCODE4 (purple network) includes genes such as CYCS, CASP3, BCL2L1, and APP. These genes are associated with apoptosis, neurodegenerative diseases, and extracellular matrix remodeling.

#### 2.3.2. GO Functional and KEGG Pathway Enrichment Analysis of Potential Targets for MZBZ Blood-Absorbable Prototype Components

**Figure 7:**
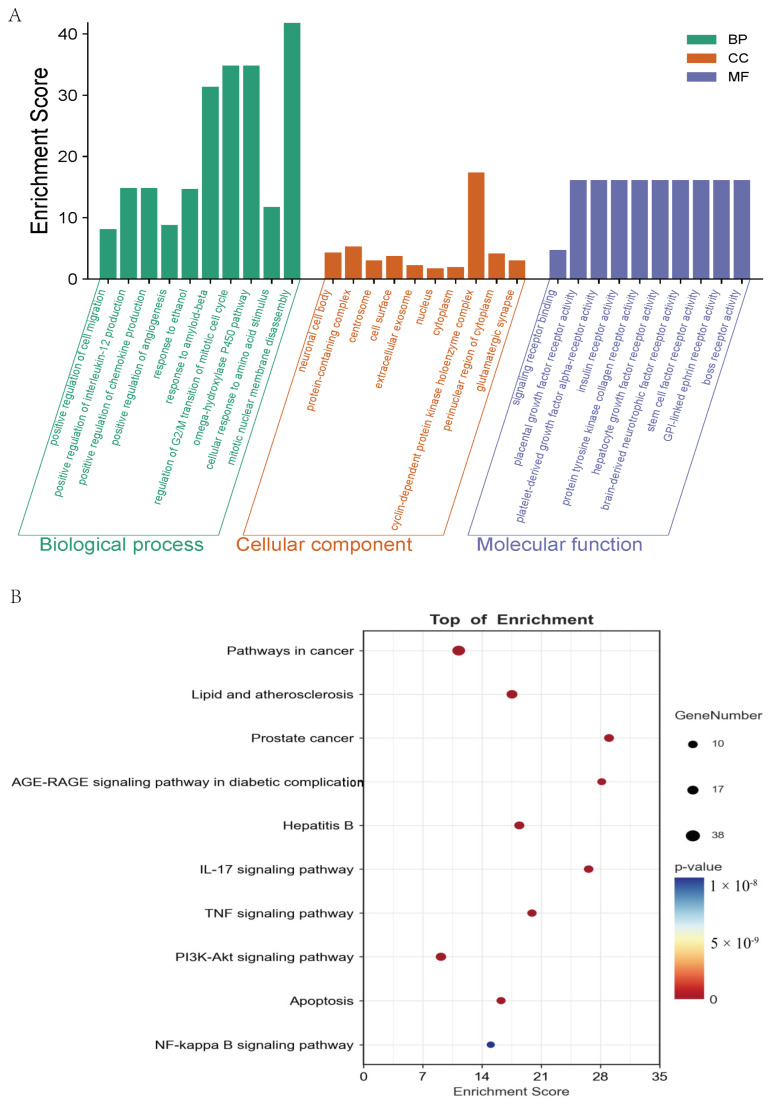
Functional enrichment analysis. (**A**) GO enrichment, biological processes (BP), cellular components (CC), and molecular functions (MF) are represented in green, orange, and blue colors, respectively. (**B**) KEGG enrichment. Bubble size indicates the number of potential targets to which the pathway belongs, and the bubble color indicates the *p*-value.

The 54 core targets obtained in Section 2.3.1 were subjected to GO and KEGG enrichment analysis. The results indicated that these targets were primarily enriched in locations such as the nucleoplasm, cytoplasm, extracellular region, cell surface, perinuclear region, and centrosome (cellular component). They involved molecular functions such as signaling receptor binding, GPIb-IX-V complex receptor activity, protein tyrosine kinase activity, platelet-derived growth factor alpha-receptor activity, insulin receptor activity, and stem cell factor receptor activity (molecular function). Furthermore, they participated in biological processes, including the positive regulation of cell migration, positive regulation of interleukin-12 production, positive regulation of chemokine production, regulation of G2/M transition, mitotic cell cycle, and response to amino acid stimulus (biological process), as shown in Figure 7A.

Pathway enrichment analysis revealed that these targets were primarily involved in pathways such as pathways in cancer, lipid and atherosclerosis, prostate cancer, AGE-RAGE signaling pathway, hepatitis B, PI3K-Akt signaling pathway, TNF signaling pathway, IL-17 signaling pathway, apoptosis, and the NF-kappa B signaling pathway, as shown in Figure 7B.

#### 2.3.3. Construction of the “MZBZ-Components-Disease-Targets-Pathways” Network

**Figure 8:**
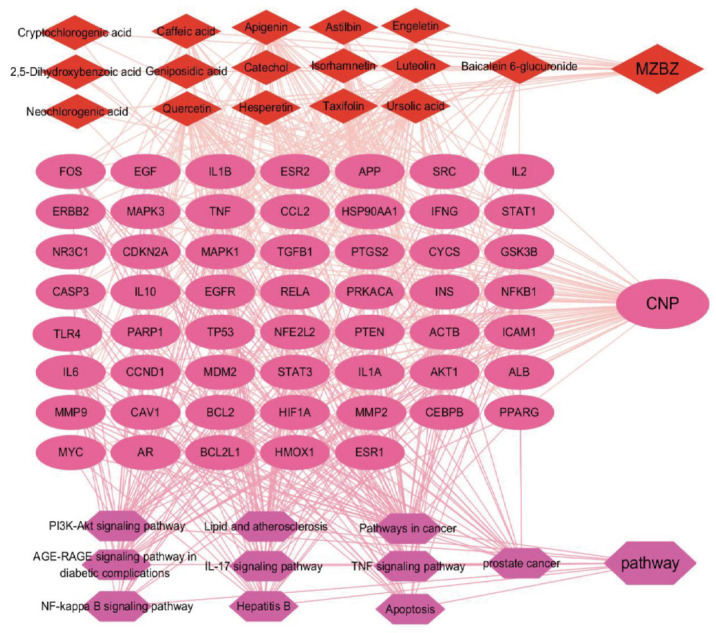
MZBZ component–target–pathway network. Note: red diamonds represent MZBZ components; purple ellipses represent core targets; dark purple hexagons represent pathways.

To deeply investigate the relationships between the blood-absorbable prototype components of MZBZ and the core targets and pathways, we meticulously constructed a network diagram of “Compound-Components-Targets-Disease-Pathways”. This network not only displays the active components in MZBZ but also details the molecular targets acted upon by these components and the related biological pathways, as shown in Figure 8. Red nodes represent effective components in the TCM compound MZBZ. Purple nodes represent key molecular targets such as NFKB1, BCL2, and COX2, and dark purple nodes represent biological pathways such as the PI3K-Akt signaling pathway, apoptosis, and the NF-kappa B signaling pathway.

MZBZ contains 16 active components. These components exert their therapeutic effects by acting on multiple key targets such as MMP2, BCL2, MMP9, IL6, and RELA, as well as through biological pathways related to inflammation, oxidative stress, and apoptosis. This discovery not only confirms the multi-target and multi-pathway mechanism of action of MZBZ but also provides an important scientific basis for further research into its therapeutic potential.

### 2.4. Docking of Key Components to Core Targets

**Figure 9:**
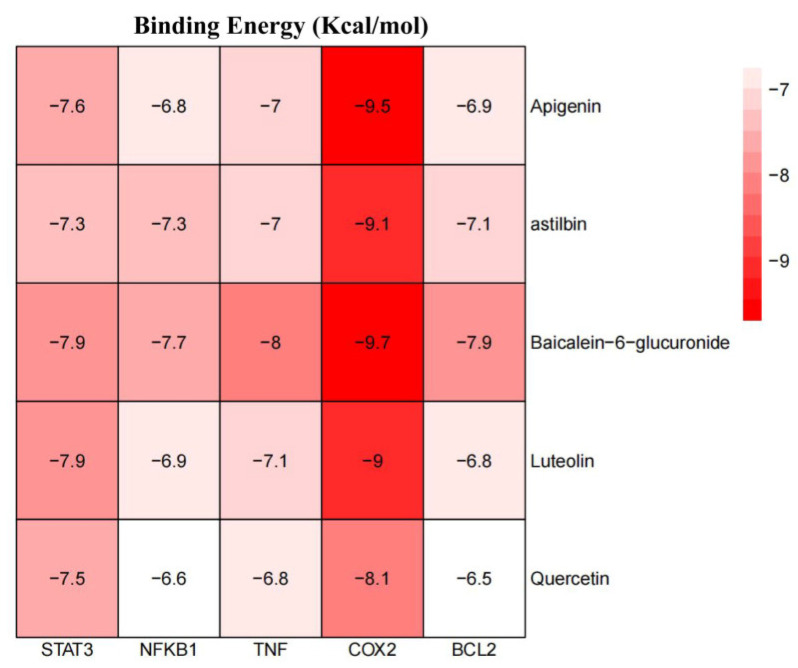
Molecular docking binding energy heatmap (kcal mol^−1^). Color gradient from light red to dark red indicates increasingly strong binding.

**Figure 10:**
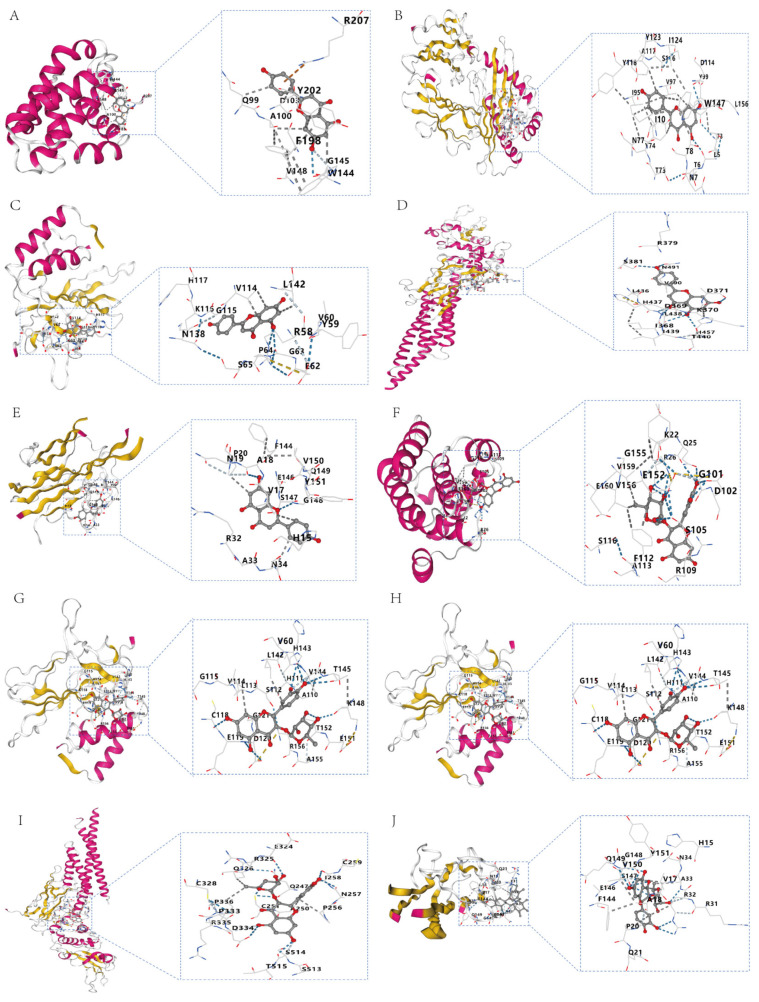

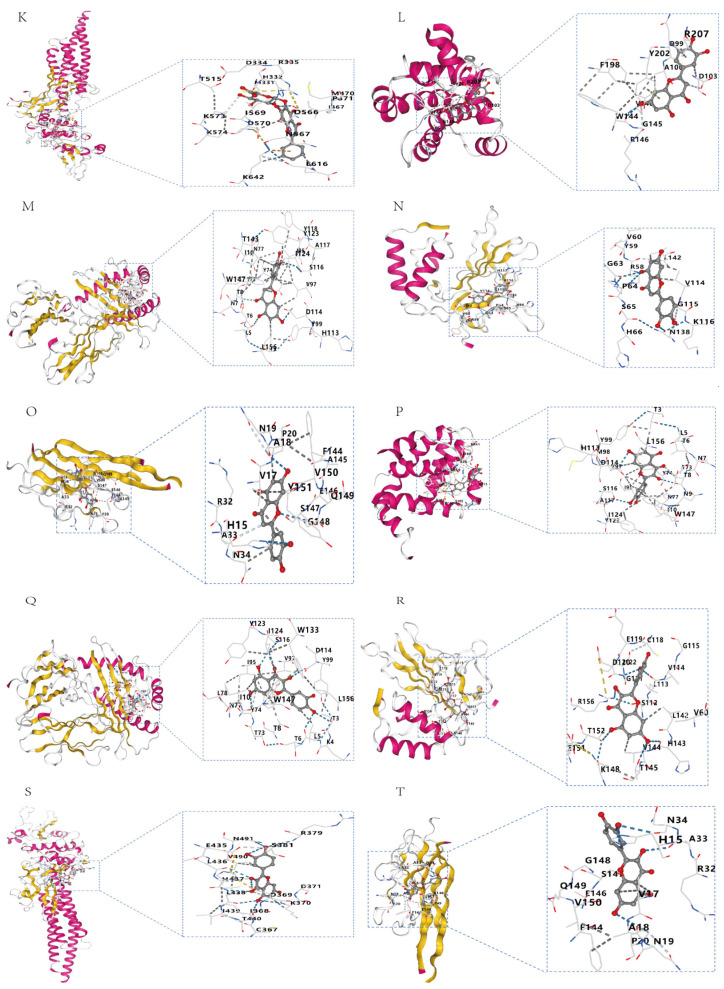
Molecular docking of key components with core targets (2D/3D). (**A**) Apigenin and Bcl2; (**B**) apigenin and COX2; (**C**) apigenin and NFκB; (**D**) apigenin and Stat3; (**E**) apigenin and TNF; (**F**) astibilin and Bcl2; (**G**) astibilin and COX2; (**H**) astibilin and NFκB; (**I**) astibilin and Stat3; (**J**) astibilin and TNF; (**K**) baicalin −6 glucuronide and Stat3; (**L**) luteolin and Bcl2; (**M**) luteolin and COX2; (**N**) luteolin and NFκB; (**O**) luteolin and TNF; (**P**) quercetin and Bcl2; (**Q**) quercetin and COX2; (**R**) quercetin and NFκB; (**S**) quercetin and Stat3; (**T**) quercetin and TNF.

To experimentally verify the inflammation-related targets predicted by network pharmacology, we employed a structure-based molecular docking approach to quantify binding affinities and characterize interaction patterns. The key top proteins (TNF, NFKB1, BCL2, STAT3, and PTGS2) were docked with the isolated compounds, yielding scores ranging from −9.7 to −6.5 kcal mol^−1^, indicative of potential modulation of inflammatory pathways (Figure 9). All compounds exhibited markedly higher binding energies for the highest-ranking target, COX2, than the remaining targets. Notably, all compounds displayed the most favorable interaction, with higher scores of −6.5 kcal mol^−1^ against each target, suggesting that this may contribute to the observed anti-inflammatory and anti-apoptotic effects by interrupting cytokine-mediated signaling. Molecular docking results for each core target with key components are shown in Figure 10.

### 2.5. In Vivo Validation

#### 2.5.1. Effects of MZBZ on Pathological Changes in the CNP Rat Model

**Figure 11:**
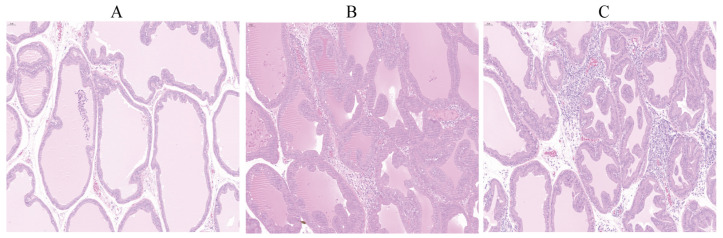
H&E histology of prostate. (**A**) Normal; (**B**) CNP model; (**C**) MZBZ. (HE 20×).

Pathological observations of rat prostate tissue showed that the prostate tissue structure in the normal group (Figure 11A) was normal, with no pathological changes such as edema or inflammatory cell infiltration. The acini were uniform in size, the prostate epithelium was of moderate thickness, the glandular lumen diameter was normal, and there was no abnormal infiltration around blood vessels. In contrast, the CNP model group (Figure 11B) exhibited significant pathological changes in the prostate tissue. There was severe diffuse inflammatory cell infiltration within the tissue, with a large number of inflammatory cells infiltrating the glandular tissue, leading to disorganized tissue structure. The acini were dilated and irregular in shape, the prostate epithelial cell layer was significantly thickened, the glandular lumen diameter was significantly reduced, and there was also obvious inflammatory cell aggregation around blood vessels and in the interstitial areas, indicating a relatively severe inflammatory response. Compared to the model group, the MZBZ intervention group (Figure 11C) showed improved pathological changes. Tissue edema and the degree of diffuse leukocyte infiltration were significantly reduced, indicating that the inflammatory response was suppressed to some extent. Although the glandular epithelium still showed mild hyperplasia, the prostate tissue structure was relatively intact, the acinar morphology was more regular, and inflammatory cell infiltration in the interstitium and around blood vessels was reduced. This suggests that MZBZ can alleviate the inflammatory response and improve histopathological damage in prostate tissue induced by CNP in rats.

#### 2.5.2. Effects of MZBZ on Changes in Serum Inflammatory Factors in CNP Rats

Based on the core targets of blood-absorbable prototype components from Section 2.3.1, targets such as IL-6, IL-17, and TNF-α were selected to detect related cytokines in serum. As shown in Table 3, Serum cytokine detection results showed that, compared to the normal group, the serum levels of IL-6, IL-17, and TNF-α were significantly increased in the model group (*p* < 0.001). After MZBZ intervention, compared to the model group, the serum levels of IL-6, IL-17, and TNF-α in rats were significantly decreased (*p* < 0.05, *p* < 0.01).

#### 2.5.3. Effects of MZBZ on the Expression of Key Targets in Prostate Tissue

**Figure 12:**
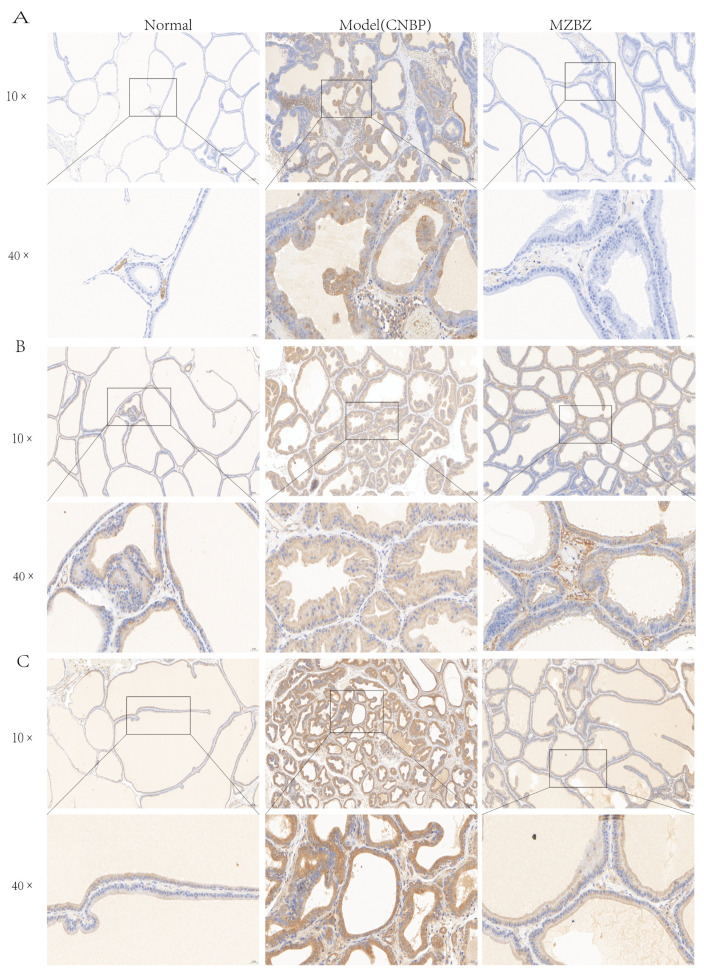
IHC of COX-2, TNF-α, and MMP-9 in prostate tissue. (**A**) COX-2; (**B**) TNA-α; (**C**) MMP-9.

Immunohistochemical profiling of COX-2 (Figure 12A), TNF-α (Figure 12B), and MMP-9 (Figure 12C) was performed on paraffin-embedded ventral prostate lobes from male SD rats with chronic nonbacterial prostatitis and subsequent MZBZ therapy. In the normal group, all three proteins exhibited only faint immunoreactivity, confined to scattered stromal fibroblasts and the single-layer epithelium. In the CNP group, markedly upregulated expression of each target (COX-2, TNF-α, MMP-9) versus the normal group was observed, with intense staining localized to infiltrating macrophages, hyperplastic epithelium, and peri-glandular smooth-muscle cells, findings consistent with chronic inflammation and tissue remodeling. Oral administration of MZBZ significantly attenuated these increments, indicating near-complete reversal of inflammation-associated molecular signatures. Representative photomicrographs of ventral prostate sections at 10× and 40× magnification are compiled in Figure 12A–C.

#### 2.5.4. Effects of MZBZ on Proteomic Changes in Prostate Tissue of the Nonbacterial Prostatitis Rat Model

**Figure 13:**
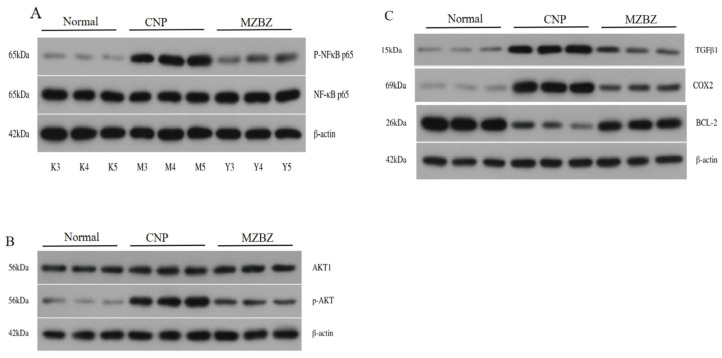
MZBZ regulates key target protein expression. Western blot image of (**A**) P-p65/p65, (**B**) p-AKT/AKT, (**C**) BCL2, COX-2, and TGF-β1 protein expression.

The effects of MZBZ on the protein expression of P-p65/p65, p-AKT/AKT, BCL2, COX-2 (PTGS2), and TGF-β1 in CNP rats were evaluated. Compared to the normal group, the expression levels of P-p65/p65, p-AKT/AKT, COX-2 (PTGS2), and TGF-β1 proteins in the prostate tissue of the model group were significantly increased (*p* < 0.001), while the BCL2 level was decreased (*p* < 0.001). Compared to the model group, the expression levels of P-p65/p65, p-AKT/AKT, COX-2 (PTGS2), and TGF-β1 proteins in the prostate tissue of rats in the various MZBZ dose groups were decreased (*p* < 0.05, *p* < 0.01), while the BCL2 level was increased, as shown in Table 4 and Figure 13.

## 3. Discussion

The pathogenesis of chronic prostatitis/chronic pelvic pain syndrome (CP/CPPS) involves a complex interplay of inflammatory and immune mechanisms, which has directed therapeutic exploration toward anti-inflammatory and immunomodulatory agents. While nonsteroidal anti-inflammatory drugs (NSAIDs) provide symptomatic relief, their long-term use is constrained by systemic toxicity [23]. In a subset of patients, autoimmune mechanisms—mediated by autoantibodies, Th17/Treg imbalance, and NLRP3-IL-1β-STAT3 signaling—play a central role in sustaining prostatic inflammation [24,25]. Genetic studies further link immune-regulatory, neurodevelopmental, and sex hormone-related loci to disease susceptibility [26], whereas epigenetic alterations such as DNA methylation and miRNA dysregulation integrate environmental influences with genetic predisposition, promoting chronicity and informing targeted therapies [27].

Neuropathic pain and neurogenic inflammation are also integral to CP/CPPS symptomatology, supporting the use of neuromodulatory agents such as tricyclic antidepressants and serotonin–norepinephrine reuptake inhibitors to attenuate pain and associated mood disturbances [28].

Beyond conventional NSAIDs, other immunomodulatory approaches have been investigated. 5α-Reductase inhibitors (e.g., finasteride) can reduce prostate volume and androgen-mediated inflammation in select patients, though predictors of response remain unclear [23]. Calcineurin inhibitors such as cyclosporine and tacrolimus may suppress refractory immune activation but carry risks of infection [29]. Monoclonal antibodies against TNF-α and natural compounds like kaempferol or Ashwagandha—which inhibit NF-κB or JAK-STAT signaling—represent promising experimental avenues [30,31]. Additionally, mesenchymal stem cells and their exosomes have shown potential to modulate IL-6/TNF-α and promote tissue repair [32,33], while synthetic hybrids such as naphthoquinone-thiazole derivatives can suppress cytokines via PI3K inhibition [34].

Collectively, current evidence underscores the multifactorial pathogenesis of CP/CPPS and highlights the need for multi-target therapeutic strategies that can concurrently address inflammatory, immune, and neuropathic components of the disease.

Traditional Chinese Medicine (TCM) has a centuries-old and continuously evolving corpus for managing prostate-related disease, as documented in classic texts and modern clinical reports. As a part of TCM, Uyghur medicine plays a vital role in the healthcare system of the Xinjiang Region of China. MZBZ is an empirical bedside formula that has been applied locally for the treatment of prostatitis for a long time. By coupling MZBZ’s clinical reputation with modern analytical platforms, the present study seeks to decode its material basis—i.e., the bioactive constituents, network targets, and pharmacological cascades—responsible for modulating CNP.

This study systematically characterized the chemical composition and in vivo blood-absorbable components of MZBZ using UHPLC-Q-Orbitrap-HRMS combined with GNPS, providing key scientific evidence for elucidating its pharmacodynamic material basis. At the level of chemical composition analysis, the study identified 188 compounds from the water extract of MZBZ, covering eight major categories, including flavonoids, organic acids, alkaloids, and phenylpropanoids, with flavonoids (79 compounds) constituting the main component group.

In the study, 35 blood-absorbable components were identified from the plasma of MZBZ-administered rats, including 18 prototype components and 17 metabolites, revealing the in vivo mode of action of this formula as “prototype-metabolite synergistic effects” [35]. The prototype components were mainly flavonoids and organic acids. The metabolites exhibited diverse structural transformations; Phase I oxidation reactions and Phase II sulfation and glucuronidation reactions significantly increased the polarity and water solubility of the components, which helps improve the in vivo exposure and target organ distribution efficiency of the drug [36]. Notably, sulfated products of flavonoid aglycones such as naringenin, apigenin, and hesperetin were detected among the metabolites. The introduction of sulfate groups may enhance the binding ability to inflammation-related enzymes, thereby strengthening the anti-inflammatory effect. Considering the pathogenesis of prostatitis, the aforementioned components may intervene in the disease process through multiple pathways; on one hand, inhibiting the inflammatory cascade triggered by bacterial infection; on the other hand, regulating chemical irritation caused by urine reflux; and simultaneously improving patient pain and psychosomatic symptoms through the neuro-endocrine-immune network.

Otherwise, among the blood-absorbed compounds, quercetin, luteolin, apigenin, isorhamnetin, and ursolic acid emerge as the most promising candidates for supporting prostate health and managing male reproductive disorders such as prostatitis, testicular damage, and prostate cancer. Studies showed that isorhamnetin selectively inhibited the growth, migration, and invasion of androgen-independent DU145 and PC3 prostate-cancer cells via induction of mitochondrial (intrinsic) apoptosis, mesenchymal–epithelial transition, suppression of MMP-2/MMP-9, and blockade of the PI3K–Akt–mTOR pathway, indicating that isorhamnetin is a promising candidate for treating androgen-independent prostate cancer [37]. Ursolic acid induces apoptosis in human prostate LNCaP cells by activating the ROCK1/PTEN pathway, elevating cofilin-1 and cytochrome c, and subsequently boosting caspase-3/9 activity, thereby suppressing cancer cell proliferation [38]. Clinical phase-I data now show that 50 mg of oral luteolin daily for 6 months is safe for men with active surveillance; tumor AR/NKX3.1 declined, and circulating miR-29/30 rose, consistent with the flavone’s activity [39]. Complementary mechanistic work in castration-resistant prostate cancer demonstrates that luteolin collapses the cytoprotective Nrf2–Keap1–Cul3 axis, selectively amplifying ROS and apoptosis in metastatic cells. Together, the studies provide the first translational evidence that luteolin can restrain early prostate cancer biology and overcome redox-adapted progression, warranting randomised evaluation [40]. It was reported that quercetin induces apoptosis, inhibits angiogenesis, and suppresses metastasis, indicating that it is a promising therapeutic tool for the treatment of prostate cancer [41]. These results indicate that compounds contained in MZBZ act through complementary mechanisms, including oxidative stress reduction, anti-inflammatory signaling, anti-prostate cancer, and modulation of androgen and apoptotic pathways.

Network pharmacology revealed that NFKB, IL-6, COX-2, IL-1β, TNF, AKT1, and BCL2 may be the key targets for MZBZ’s anti-CNP effects, while the PI3K-Akt signaling pathway, apoptosis, and NF-kappa B signaling pathway may be potential pathways for MZBZ’s prevention and treatment of CNP. Levels of TNF-α, IL-1β, and IL-6 are significantly elevated in the prostatic fluid, serum, or tissues of chronic patients [42]. In the experimental autoimmune prostatitis (EAP) model, these cytokines are also significantly upregulated, suggesting their core role in non-infectious inflammation [43]. The NF-κB and PI3K/AKT signaling pathways play a central role in the pathogenesis of chronic prostatitis and chronic pelvic pain syndrome, primarily driving disease progression through inflammation activation, immune dysregulation, cell proliferation, and anti-apoptotic mechanisms. NF-κB is activated in chronic prostatitis by pro-inflammatory cytokines (TNF-α, IL-1β) or oxidative stress, inducing the expression of inflammatory mediators such as IL-6, IL-8, and COX-2, leading to sustained inflammation in prostate tissue 22. NF-κB activation promotes macrophage polarization toward the M1 type, releasing inflammatory factors and stimulating nerve endings within the prostate, leading to pain and urinary abnormalities [44]. PI3K/AKT, activated by inflammatory factors (such as IL-4, IGF-1), inhibits apoptosis and promotes fibroblast proliferation, participating in prostate stromal fibrosis. Simultaneously blocking both NF-κB and PI3K/AKT pathways (e.g., using IKK inhibitor + AKT inhibitor) may be more effective at alleviating chronic prostatitis. This study found that MZBZ Wan can reduce the levels of inflammatory factors IL-1β, IL-6, and TNF-α in the prostate tissue of CNP rats. MZBZ can inhibit the activation of the NF-κB signaling pathway, PI3K/AKT, and the expression of apoptosis-related proteins, suggesting that MZBZ can improve CNP-related symptoms through multiple targets and pathways.

The current trajectory in chronic nonbacterial prostatitis (CNP) research reflects a paradigm shift toward identifying and targeting novel mechanistic pathways, particularly those involving the neuroimmune axis, specific ion channels, and the pelvic microbiome. Emerging pharmacological agents directed at these targets hold promise for addressing the underlying pathophysiology rather than merely alleviating symptoms. However, most of these innovative therapies remain in the nascent stages of development, and rigorous clinical validation through well-designed trials is imperative to establish their safety, efficacy, and applicability across diverse patient subgroups.

In our study, although preliminary in vivo evidence was obtained, the network-predicted targets and pathways require further validation. Broader and deeper experimental confirmation is needed, particularly through a multi-omics approach to fully elucidate the anti-inflammatory, immunomodulatory, and gut microbiome regulatory mechanisms of MZBZ. Systematic clinical studies are also warranted to confirm its efficacy. Future work should expand in vitro and in vivo investigations of these targets, integrate comprehensive pharmacokinetic profiling of MZBZ, and employ multi-omics combined with randomized controlled trials to establish the clinical translatability of the identified metabolite signature and to define optimal dose–exposure–response relationships.

In conclusion, this study clarified the material basis and in vivo action forms of MZBZ, conducted in-depth verification of the component–target–pathway network, and evaluated related targets and pathways by combining molecular docking, network pharmacology, and animal experiments, further revealing the mechanism of action of MZBZ and providing more comprehensive scientific support for the precise clinical use of MZBZ and the construction of a quality control system.

## 4. Materials and Methods

### 4.1. Chemicals, Reagents, and Instruments

An ultra-high-performance liquid chromatography system (Dionex Ultimate 3000, Bremen, Germeny) hyphenated to a Q-Exactive Orbitrap high-resolution mass spectrometer (Thermo Fisher Scientific, Waltham, MA, USA) was used for all analyses. Additional instruments included an AE224C analytical balance (Shanghai Sunny Hengping Scientific Instrument Co., Ltd., Shanghai, China), a TGL-16 high-speed refrigerated benchtop centrifuge (Hangzhou Deju Instrument Equipment Co., Ltd., Hangzhou, China), and a KQ-250B ultrasonic cleaner (Kunshan Ultrasonic Instrument Co., Ltd., Kunshan, China). LC–MS-grade methanol, acetonitrile, formic acid, and water were obtained from Thermo Fisher Scientific (USA). Reference standards were as follows: apigenin (Batch No. 19070308) and astragalin (Batch No. 13062403) from Shanghai PureOne Biotechnology Co., Ltd. (Shanghai, China); cryptochlorogenic acid (Batch No. 180324035) from the Beijing Beina Chuanglian Biotech Research Institute (Beijing, China); luteolin-7-O-glycoside (Batch No. 151111) from Chengdu Pufei De Biotech Co., Ltd. (Chengdu, China); rutin (Batch No. 100080-201811), luteolin (Batch No. 11520-200504), and quercetin (Batch No. 100081201408) from the National Institutes for Food and Drug Control (Beijing, China); and kaempferol-3-O-rutinoside (Batch No. WP24080702) from Sichuan Weikeqi Biotechnology Co., Ltd. (Chengdu, China). Antibodies and kits included a BCA protein assay kit (Cat. No. WLA004, Wanleibio, Shenyang, China) and primary antibodies against p-NF-κB p65 (WL02169), NF-κB p65 (WL01980), BCL2 (WL01556), COX-2 (WL01750), AKT1 (WL01652), p-AKT (WLP001), TGF-β1 (WL02193), and β-actin (WL01372), together with HRP-conjugated goat anti-rabbit IgG (WLA023; all from Wanleibio, Shenyang, Chnia).

### 4.2. Ethics

All animal procedures complied with institutional guidelines and were approved by the IACUC of Xinjiang Medical University (IACUC-JT-20230321-28).

### 4.3. Preparation of MZBZ and Reference Solutions

Crude MZBZ (prescription ratio as per pharmacopeial practice) was extracted twice by reflux with 70% ethanol (10×, 1 h each), filtered, concentrated, and reconstituted (2 g per 50 mL). Powdered MZBZ (100-mesh, 4.0 g) for direct testing was sonicated in 70% ethanol (80 mL, 30 min, 700 W/40 kHz), centrifuged (14,000 rpm), and filtered (0.22 µm). Mixed standards were dissolved in methanol (1 mg·mL^−1^ each).

### 4.4. Serum Pharmacochemistry

#### 4.4.1. Drug-Containing Serum

Male SD rats (200 ± 20 g; SPF) were fasted for 12 h with water ad libitum, then administered MZBZ by gavage (10 g·kg^−1^ crude-equivalent). Blood was collected (15 min–8 h) into EDTA tubes; plasma was separated (2400× *g*, 10 min) and stored at −80 °C.

#### 4.4.2. Plasma Processing

Plasma (100 µL) was deproteinated (acetonitrile 300 µL), vortexed (1 min), centrifuged (18,900× *g*, 7 min), evaporated (N_2_, 40 °C), reconstituted (100 µL methanol), sonicated (1 min), cleared by centrifugation, and injected (5 µL).

### 4.5. UHPLC-Q-Orbitrap-HRMS

Column: Waters ACQUITY UPLC HSS T3 (100 × 2.1 mm, 1.7 µm) at 35 °C. Mobile phases: A = 0.1% formic acid in water; B = acetonitrile. Gradient: 0–5 min 5–15% B; 5–15 min 15–20% B; 15–25 min 20–55% B; 25–30 min 55–100% B; flow 0.25 mL·min^−1^; injection 2 µL. ESI±: spray 3.0 kV/−2.5 kV; source/capillary 320/350 °C; sheath/aux gas 40/10 a.u. Full MS/dd-MS^2^, *m*/*z* 100–1500, 70,000/17,500 resolution; NCE 20/40/60 eV.

### 4.6. Data Processing and GNPS

A self-built database integrated MassBank, ChemSpider, PubChem, and mzCloud. Compound Discoverer 3.3 performed feature alignment and library matching. GNPS molecular networking used precursor/fragment tolerances of 0.02 Da, cosine ≥0.7, ≥5 matched fragments, maximum mass shift of 700 Da, TopK = 15, β = 100. Redundant nodes were merged and visualized.

### 4.7. Network Pharmacology

#### 4.7.1. Predicting Putative Anti-Inflammatory Targets

The 18 blood absorbable compounds were first uploaded to PubChem to retrieve their canonical SMILES and used for supplementary prediction of interactions (probability > 0) via SwissTargetPrediction and ETCM (http://www.tcmip.cn/ETCM/ (accessed on 25 July 2024) for target prediction (confidence index > 0.8). Targets from these databases were integrated, deduplicated, and standardized to establish a component–target database.

#### 4.7.2. Network Construction and Enrichment Analysis

In parallel, chronic nonbacterial prostatitis-related genes were harvested from GeneCards and OMIM.ORG. Targets with a relevance score ≥ 20 were retained and deduplicated. Common targets between drug components and disease were identified using Venny 2.1. The intersection of compound- and disease-derived targets was imported into STRING (https://string-db.org/ (accessed on 28 July 2024) and filtered with the default high-confidence settings: organism “Homo sapiens”, interaction score ≥ 0.7, and FDR ≤ 5%. The output network was constructed using Cytoscape 3.10.2 for visualization and to establish the PPI network. Key targets were determined using CentiScape2.2. Module analysis was performed using Metascape (http://metascape.org (accessed on 24 June 2025). The functional enrichment analysis (GO and KEGG) of key therapeutic targets was conducted using DAVID (*p* < 0.05, FDR < 0.05), and the top 10 terms were visualized.

### 4.8. Molecular Docking

Representative plasma-exposed flavonoids were docked to COX-2 (PTGS2), NFKB1, TNF, IL-6, and BCL2 using standard protocols (protein preparation, grid definition at active sites, exhaustiveness set per target). Binding energies/poses were summarized as 2D/3D interaction maps. Briefly, the preprocessing step, carried out in AutoDock Tools 1.5.6, involved hydrogen completion, charge attribution, and grid-box setup, whereas the protein models were downloaded directly from the Protein Data Bank (https://www.rcsb.org (accessed on 1 September 2025). The compound structures were obtained from PubChem (https://pubchem.ncbi.nlm.nih.gov (accessed on 1 September 2025).

### 4.9. Experimental Validation

#### 4.9.1. CNP Model and Treatment

Five- to six-week-old male Sprague–Dawley SPF rats (180 ± 20 g) supplied by Xinjiagn Medical University Animal Center, Urumqi, China (license SYSK [Xin] 2020–003), were group-housed (4–5 per cage) with unrestricted chow and water under 24 °C and a 12 h light/dark cycle. 24 animals were randomized into 3 sets (8 each): Normal, Model, and MZBZ treated groups. Under pentobarbital anesthesia (100 mg·kg^−1^, i.p.), the dorsal prostate lobes were exposed; Model/MZBZ received bilateral injections of 1% λ-carrageenan (250 µL/lobe); Normal group received saline. After 7 days, MZBZ was administered via gavage (14 g·kg^−1^·day^−1^; decoction concentrated to 3.5 g·mL^−1^) for 6 weeks; controls received saline.

#### 4.9.2. Sample Collection and Histology

Twenty-four hours post-final dose, blood (abdominal aorta) and prostate lobes were collected. Left lobes were fixed (4% formaldehyde), paraffin-embedded, sectioned (4 µm), and H&E-stained; right lobes were snap-frozen for protein assays.

#### 4.9.3. Immunohistochemistry

Antigen retrieval (10 mM citrate, pH 6.0, 15 min) followed deparaffinization. Endogenous peroxidase was quenched (3% H_2_O_2_, 10 min); sections were blocked (5% goat serum, 30 min) and incubated at 4 °C overnight with COX-2 (1:200, ab15191), TNF-α (1:150, ab183218), or MMP-9 (1:200, ab76003). Biotinylated goat anti-rabbit IgG (1:500) was applied; detection used DAB and hematoxylin counterstain. H-score (0–300) was calculated from five random 400× fields by a blinded observer.

#### 4.9.4. Cytokines and Western Blot

Serum IL-6, IL-17, TNF-α, and tissue MMP-2/MMP-9 were measured using ELISA. For Western blot, tissue lysates (RIPA + protease/phosphatase inhibitors) were quantified (BCA), resolved (10% SDS-PAGE), and transferred to PVDF. Membranes were blocked (5% milk) and probed (1:1000) for p-p65, p65, COX-2, AKT1, p-AKT1, TGF-β1, and BCL2 (β-actin as loading control). ECL signals were quantified using ImageJ 2x.

### 4.10. Statistics

Data are shown as mean ± SD. One-way ANOVA with appropriate post hoc tests was conducted in GraphPad Prism 8.0. Significance was defined as *p* < 0.05.

## 5. Conclusions

The present study integrates chemical profiling, metabolite identification, network pharmacology, molecular docking, and in vivo validation to clarify the substance basis and mechanism of Maizibizi Wan (MZBZ) against CNP. We demonstrate that a complex of 35 blood-accessible prototypes and their Phase-I/II metabolites—rather than any single compound—accounts for the efficacy we observed. These constituents converge on 54 core targets, notably NF-κB and PI3K/AKT hubs, to suppress TNF-α/IL-1β/IL-6/IL-17 release, down-regulate COX-2/TGF-β1, restore BCL2-mediated apoptosis balance, and ultimately ameliorate prostate inflammation; molecular docking corroborates high-affinity binding of key flavonoids to COX-2, NFKB1, TNF, and BCL2, supplying structural plausibility for the network predictions. Together, the results provide a mechanistic rationale for the clinical use of MZBZ and indicate that plasma-exposed flavonoid metabolites are marker candidates for batch-to-batch quality control.

Although preliminary in vivo evidence was obtained, the network-predicted targets and pathways remain insufficiently validated; broader and deeper experimental confirmation is required. Future work should expand both in vitro and in vivo interrogations of these targets, integrate comprehensive pharmacokinetic profiling of MZBZ, and employ multi-omics coupled with randomized controlled trials to establish the clinical translatability of the identified metabolite signature and to define optimal dose–exposure–response relationships.

## Figures and Tables

**Table 1:** Characterization of chemical components of MZBZ.

No.	Identification	*t*_R_/min	MS Fragment	*m*/*z*	Formula	Ion Type	Error/ppm	Classification	Resource
1	Gluconic acid	0.96	177(5), 129(29), 75(100)	195.0511	C_6_H_12_O_7_	[M−H]^−^	0.46	Organic acid	[16,17,18,19,20]
2	Proline	1.01	87(3), 70(100)	116.0707	C_5_H_9_NO_2_	[M+H]^+^	0.26	Other	[16,17,18,19,20]
3	Citric acid	1.09	111(100), 87(56), 85(32), 57(9)	191.0201	C_6_H_8_O_7_	[M−H]^−^	1.45	Organic acid	[16,17,18,19,20]
4	Malic acid	1.17	115(19), 85(43), 71(56)	133.0142	C_4_H_6_O_5_	[M−H]^−^	1.75	Organic acid	[16,17,18,19,20]
5	Methylmalonic acid or isomer	1.70	73(100)	117.0196	C_4_H_6_O_4_	[M−H]^−^	1.75	Organic acid	[16,17,18,19,20]
6	Galloyl hexoside	2.00	169(100), 125(62)	331.0679	C_13_H_16_O_10_	[M−H]^−^	4.07	Organic acid	[16,17,18,19,20]
7	Furoic acid	2.33	67(81)	111.0091	C_5_H_4_O_3_	[M−H]^−^	2.03	Organic acid	[16,17,18,19,20]
8	Citric acid methyl ester	2.34	143(10), 111(100), 87(47)	205.0357	C_7_H_10_O_7_	[M−H]^−^	1.38	Organic acid	[16,17,18,19,20]
9	Dihydroxybenzoic acid hexoside or isomer	2.67	153(100), 109(7)	315.0731	C_13_H_16_O_9_	[M−H]^−^	4.22	Organic acid	[16,17,18,19,20]
10	Phenylalanine	2.97	149(3), 120(100), 103(10)	166.0862	C_9_H_11_NO_2_	[M+H]^+^	1.11	Alkaloid	[16,17,18,19,20]
11	Methylmalonic acid or isomer	3.30	87(100)	131.0352	C_5_H_8_O_4_	[M−H]^−^	2.36	Organic acid	[16,17]
12	Protocatechuic acid	3.39	109(13)	153.0197	C_7_H_6_O_4_	[M−H]^−^	2.28	Organic acid	[16,17]
13	Dihydroxybenzoic acid hexoside or isomer	3.45	153(100), 109(64)	315.0725	C_13_H_16_O_9_	[M−H]^−^	4.41	Organic acid	[16,17,18,19,20]
14	Vanillic acid	3.80	152(100), 123(26), 108(48)	167.0353	C_8_H_8_O_4_	[M−H]^−^	0.29	Organic acid	[16,17,18,19,20]
15	Geniposidic acid	3.90	211(32), 193(8), 167(22), 149(51), 123(100)	373.1135	C_16_H_22_O_10_	[M−H]^−^	−0.26	Organic acid	[16,17]
16	Gentisic acid	4.08	109(100)	153.0197	C_7_H_6_O_4_	[M−H]^−^	0.7	Organic acid	[16,17,18,19,20]
17	Kojic acid	4.45	97(3), 69(10)	143.0338	C_6_H_6_O_4_	[M+H]^+^	−1.47	Organic acid	[16,20]
18	Caffeoylquinic acid–hexoside or isomer	4.52	353(16), 191(100), 179(13), 135(14)	515.1422	C_22_H_28_O_14_	[M−H]^−^	2.24	Organic acid	[20]
19	Tryptophan	4.85	188(100), 146(95), 144(21), 132(10), 118(41)	205.0968	C_11_H_11_NO_2_	[M+H]^+^	−1.52	Other	[16,17,18,19,20]
20	caffeoylquinic acid–hexoside or isomer	5.59	353(12), 191(100), 179(10), 135(12)	515.1414	C_22_H_28_O_14_	[M−H]^−^	3.66	Organic acid	[20,21]
21	Ferulic acid–hexoside or isomer	5.87	193(100), 178(13), 149(23), 134(80)	355.1042	C_16_H_20_O_9_	[M−H]^−^	3.24	Organic acid	[16,17,18,19,20]
22	Caffeoylquinic acid–hexoside or isomer	6.14	353(12), 191(100), 179(16), 135(20)	515.1415	C_22_H_28_O_14_	[M−H]^−^	2.47	Organic acid	[20,21]
23	Scopoletin	6.29	178(21), 133(34)	193.0492	C_10_H_8_O_4_	[M+H]^+^	−2.04	Phenylpropanoids	[20]
24	Methylquinoline	6.39	117(4), 103(3)	144.0806	C_9_H_8_NO	[M+H]^+^	−1.57	Alkaloid	[16,17,18,19,20]
25	Solanigrine A or isomer	6.50	930(27), 912(100), 894(12), 85(12)	930.4670	C_45_H_71_NO_19_	[M+H]^+^	−2.44	Alkaloid	[21]
26	Quercetin–dihexoside or isomer	6.62	463(43), 301(38), 299(100), 271(47), 243(5), 151(7), 121(6)	625.1420	C_27_H_30_O_17_	[M−H]^−^	1.23	Flavonoid	[20]
27	Vanillin or isomer	6.62	136(2), 109(19)	151.0404	C_8_H_8_O_3_	[M−H]^−^	0.22	Organic acid	[16,17,18,19,20]
28	Procyanidin B2	6.73	577(5), 451(10), 425(17), 407(46), 289(67), 255(7), 161(22), 125(100)	577.1362	C_30_H_26_O_12_	[M−H]^−^	1.54	Flavonoid	[16]
29	Esculetin	6.79	149(4), 133(27), 105(15), 89(2)	177.0194	C_9_H_6_O_4_	[M−H]^−^	0.19	Phenylpropanoids	[16,17,18,19,20].
30	CQA–hexoside or isomer	7.06	353(4), 191(100), 179(4), 135(3)	515.1416	C_22_H_28_O_14_	[M−H]^−^	4.02	Organic acid	[20,21]
31	Ferulic acid–hexoside or isomer	7.17	193(100), 178(33), 149(16), 134(55)	355.1039	C_16_H_20_O_9_	[M−H]^−^	2.75	Organic acid	[16,17,18,19,20]
32	Sibiricose A1	7.37	323(3), 285(13), 223(100), 205(93), 190(88), 175(26), 164(19), 149(23)	547.1679	C_23_H_32_O_15_	[M−H]^−^	1.83	Other	[20]
33	Cryptochlorogenic acid	7.37	191(52), 179(67), 173(100), 135(71), 93(27), 85(9)	353.0882	C_16_H_18_O_9_	[M−H]^−^	0.92	Organic acid	[20,21]
34	Pyranamide C	7.44	472(100), 310(15), 220(37), 163(54), 145(5)	472.2431	C_25_H_33_N_3_O_6_	[M+H]^+^	−0.75	Alkaloid	[20]
35	Syringic acid	7.44	155(19), 140(100), 125(12)	197.0459	C_9_H_10_O_5_	[M−H]^−^	1.16	Organic acid	[16]
36	Kaempferol 3-(2G-glucosylrutinnoside)-7-glucoside	7.70	917(8), 771(100), 429(5), 285(23), 284(47), 255(37), 227(15), 151(5)	917.2592	C_39_H_50_O_25_	[M−H]^−^	2.59	Flavonoid	[21,22]
37	Quercetin 3-(2G-glucosylrutinoside)	7.74	771(95), 300(100), 271(66), 255(37), 243(14), 179(5), 151(7)	771.2006	C_33_H_40_O_21_	[M−H]^−^	1.93	Flavonoid	[17,21]
38	Okanin–dihexose or isomer	8.22	611(5), 449(75), 287(82), 151(100), 135(53), 107(18)	611.1630	C_27_H_32_O_16_	[M−H]^−^	1.97	Flavonoid	[20]
39	Parmosidone G	8.27	473(3), 411(36), 381(20), 351(45), 309(54), 163(44), 145(100), 119(36)	507.1281	C_27_H_24_O_10_	[M−H]^−^	−3.06	Other	[21]
40	Theogallin	8.36	343(5), 281(3), 197(14), 145(100), 119(67)	343.0677	C_14_H_16_O_10_	[M−H]^−^	1.89	Organic acid	[16]
41	Cinchonain IIa	8.38	739(41), 587(32), 449(21), 435(10), 339(30), 289(51), 177(100), 161(24), 137(27)	739.1690	C_39_H_32_O_15_	[M−H]^−^	2.84	Other	[16]
42	Luteolin–dihexoside or isomer	8.52	447(100), 285(90), 284(37), 151(2), 133(2)	609.1473	C_27_H_30_O_16_	[M−H]^−^	3.72	Flavonoid	[20]
43	Apigenin–dihexoside	8.53	595(5), 433(31), 271(100), 153(4)	595.1643	C_27_H_30_O_15_	[M+H]^+^	−2.53	Flavonoid	[20]
44	Taxifolin–hexoside or isomer	8.56	465(70), 303(77), 285(75), 179(15), 151(14), 125(100)	465.1046	C_21_H_22_O_12_	[M−H]^−^	1.71	Flavonoid	[16,17,18,19,20]
45	Feruloylquinic acid or isomer	8.62	191(100), 173(10), 134(12), 93(29)	367.1041	C_17_H_20_O_9_	[M−H]^−^	3.32	Organic acid	[20,21]
46	Vanillin or isomer	8.73	136(100), 108(7)	151.0403	C_8_H_8_O_3_	[M−H]^−^	0.29	Organic acid	[16,17,18,19,20]
47	Quercetin–dihexoside or isomer	8.80	463(100), 301(84), 300(66), 299(7), 271(34), 255(13), 243(5), 151(23)	625.1421	C_27_H_30_O_17_	[M−H]^−^	3.46	Flavonoid	[22]
48	Alternatain D	8.93	319(25), 233(73), 173(42), 163(21), 145(32), 119(24)	379.1043	C_18_H_20_O_9_	[M−H]^−^	2.25	Other	[21]
49	Kaempferol–dihexoside or isomer	9.01	447(9), 285(16), 284(20), 283(94), 255(49), 151(4)	609.1476	C_27_H_30_O_16_	[M−H]^−^	4.22	Flavonoid	[22]
50	Solanigrine A or isomer	9.02	930(100), 912(12), 894(13), 85(10)	930.4671	C_45_H_71_NO_19_	[M+H]^+^	−2.37	Alkaloid	[21]
51	Okanin–dihexose or isomer	9.24	449(80), 287(68), 151(100), 135(71), 107(15)	611.1628	C_27_H_32_O_16_	[M−H]^−^	1.67	Flavonoid	[20]
52	Quercetin–dihexoside or isomer	9.36	303(100), 229(4), 127(2)	627.1539	C_27_H_30_O_17_	[M+H]^+^	−2.72	Flavonoid	[21,22]
53	Solanigroside Q or isomer	9.46	916(100), 898(15), 880(6), 85(13)	916.4881	C_45_H_73_NO_18_	[M+H]^+^	−2.12	Alkaloid	[21]
54	Taxifolin or isomer	9.56	305(99), 287(29), 259(77), 231(58), 213(11), 153(100), 149(72), 123(67)	305.0649	C_15_H_12_O_7_	[M+H]^+^	−2.2	Flavonoid	[16]
55	Plantagoside	9.59	465(100), 303(68), 297(51), 166(54), 153(51), 135(82)	465.1046	C_21_H_22_O_12_	[M−H]^−^	1.64	Flavonoid	[16,17,21,22]
56	Suberic acid	9.67	111(100), 83(13)	173.0821	C_8_H_14_O_4_	[M−H]^−^	1.04	Organic acid	[16,17,18,19,20]
57	Rimocidin	9.71	768(100), 750(14), 732(18), 85(10)	768.4151	C_39_H_61_NO_14_	[M+H]^+^	−1.8	Alkaloid	[21]
58	Solanigrine A or isomer	9.71	930(100), 912(12), 894(11), 85(12)	930.4673	C_45_H_71_NO_19_	[M+H]^+^	−2.17	Alkaloid	[21]
59	Quercetin–deoxyhexosie–hexoside or isomer	9.74	301(18), 300(100), 271(50), 179(2), 151(6)	609.1473	C_27_H_30_O_16_	[M−H]^−^	3.82	Flavonoid	[19,21]
60	Feruloylquinic acid or isomer	9.91	191(100), 173(10), 135(100)	367.104	C_17_H_20_O_9_	[M−H]^−^	3.47	Organic acid	[19,21]
61	Cinchonain IIb	9.96	739(25), 587(30), 449(17), 435(12), 339(32), 289(43), 177(100), 161(26), 137(18)	739.1684	C_39_H_32_O_15_	[M−H]^−^	1.88	Other	[16]
62	Kaempferol–dihexoside or isomer	10.01	609(15), 447(95), 285(100), 284(28), 255(8), 151(2), 133(3)	609.1469	C_27_H_30_O_16_	[M−H]^−^	0.94	Flavonoid	[19,20,21]
63	Luteolin–dihexoside or isomer	10.04	447(100), 285(100), 284(27), 255(8), 151(3), 133(2)	609.1468	C_27_H_30_O_16_	[M−H]^−^	2.12	Flavonoid	[20]
64	Solanigroside Q or isomer	10.10	916(100), 898(14), 880(17), 85(8)	916.4885	C_45_H_73_NO_18_	[M+H]^+^	−1.71	Alkaloid	[21]
65	Solanigrine A or isomer	10.31	930(100), 912(22), 894(10), 85(14)	930.4667	C_45_H_71_NO_19_	[M+H]^+^	−2.83	Alkaloid	[21]
66	Isovitexin 2″-O-feruloyl-glucoside-4′-O-glucoside	10.44	929(14), 769(75), 315(100), 314(49), 300(32), 299(30), 271(22), 243(12)	931.2543	C_43_H_48_O_23_	[M−H]^−^	3.19	Flavonoid	[21]
67	Taxifolin–hexoside or isomer	10.49	465(46), 303(24), 151(100), 107(12)	465.1044	C_21_H_22_O_12_	[M−H]^−^	1.17	Flavonoid	[20]
68	Ferulic acid or isomer	10.52	178(66), 149(23), 134(100)	193.0509	C_10_H_10_O_4_	[M−H]^−^	0.76	Organic acid	[20,22]
69	Feruloyloctopamine or isomer	10.55	328(6), 311(19), 310(100), 161(91), 133(61)	328.1198	C_18_H_19_NO_5_	[M−H]^−^	2.42	Alkaloid	[20,21]
70	Kaempferol–dihexoside or isomer	10.56	447(100), 285(44), 284(67), 255(52), 227(29), 151(5)	609.1475	C_27_H_30_O_16_	[M−H]^−^	4.12	Flavonoid	[21]
71	Sophoraflavonloside	10.58	609(100), 447(10), 285(39), 284(60), 255(50), 227(29), 151(5), 135(3)	609.1476	C_27_H_30_O_16_	[M−H]^−^	2.17	Flavonoid	[20,21]
72	Luteolin-7-O-glucoside	10.59	285(100), 284(59), 151(5), 133(9)	447.0942	C_21_H_20_O_11_	[M−H]^−^	4.51	Flavonoid	[16,22]
73	Solanigroside Q or isomer	10.71	916(100), 898(12), 880(17), 85(7)	916.4883	C_45_H_73_NO_18_	[M+H]^+^	−1.86	Alkaloid	[21]
74	Coumaric acid hexoside or isomer	10.84	163(100), 135(17), 117(7), 97(14)	325.0908	C_15_H_16_O_7_	[M+H]^+^	−2.24	Phenylpropanoids	[20]
75	Quercetin-deoxyhexosie–hexoside or isomer	10.86	301(41), 300(74), 271(39), 179(4), 151(19)	609.1472	C_27_H_30_O_16_	[M−H]^−^	3.62	Flavonoid	[21,22]
76	Solanigrine B or isomer	10.91	914(100), 897(6), 896(12), 878(13), 85(12)	914.4734	C_45_H_71_NO_18_	[M+H]^+^	−1.1	Alkaloid	[21]
77	Plantamajoside isomer	10.96	639(44), 477(16), 179(2), 161(100), 135(12), 133(26)	639.1940	C_29_H_36_O_16_	[M−H]^−^	1.42	Phenylethanoid glycoside	[20]
78	Sinaticin	11.14	435(21), 393(11), 325(62), 313(62), 283(14), 163(29), 123(100)	435.1064	C_24_H_18_O_8_	[M+H]^+^	−2.37	Flavonoid	[16]
79	Rutin	11.20	609(100), 301(36), 300(77), 271(48), 255(23), 243(8), 179(5), 151(12), 107(4)	609.1472	C_27_H_30_O_16_	[M−H]^−^	1.54	Flavonoid	[19]
80	Solanigrine B or isomer	11.29	914(100), 897(5), 896(13), 878(13), 85(10)	914.4726	C_45_H_71_NO_18_	[M+H]^+^	−1.97	Alkaloid	[21]
81	Sinensin	11.33	341(36), 287(87), 217(11), 189(19), 151(100), 135(71), 107(15)	449.1095	C_21_H_22_O_11_	[M−H]^−^	1.09	Flavonoid	[19,20]
82	Solanigrine D or isomer	11.34	754(100), 736(15), 718(19), 85(8)	754.4364	C_39_H_63_NO_13_	[M+H]^+^	−1.08	Alkaloid	[21]
83	Hyperoside	11.38	301(100), 300(20), 271(2), 255(2), 151(32), 107(9)	463.0891	C_21_H_20_O_12_	[M−H]^−^	4.27	Flavonoid	[20,22]
84	Ferulic acid or isomer	11.41	178(26), 149(11), 134(100)	193.0508	C_10_H_10_O_4_	[M−H]^−^	1.08	Organic acid	[19,21]
85	Procyanidin B4	11.49	577(7), 451(11), 425(17), 407(37), 289(49), 161(23), 151(10), 125(100)	577.1361	C_30_H_26_O_12_	[M−H]^−^	1.29	Flavonoid	[16]
86	Solanigroside Q or isomer	11.50	916(100), 898(3), 85(15)	916.4872	C_45_H_73_NO_18_	[M+H]^+^	−3.12	Alkaloid	[21]
87	Astilbin or isomer	11.55	303(18), 285(40), 179(20), 151(100), 125(24), 107(20)	449.1095	C_21_H_22_O_11_	[M−H]^−^	3.62	Flavonoid	[16]
88	Isorhamnetin–dihexoside	11.68	639(100), 315(36), 314(31), 300(19), 299(33), 285(6), 271(26), 255(10), 243(20), 151(3)	639.1580	C_28_H_32_O_17_	[M−H]^−^	1.77	Flavonoid	[21]
89	Sinapinic acid	11.73	208(36), 193(26), 164(56), 149(100), 121(32)	223.0615	C_11_H_12_O_5_	[M−H]^−^	1.41	Organic acid	[21]
90	Quercetin–hexoside or isomer	11.89	463(69), 301(53), 300(100), 271(70), 255(31), 243(15), 179(5), 151(21)	463.0894	C_21_H_20_O_12_	[M−H]^−^	2.15	Flavonoid	[16,17,18,19,20]
91	Luteolin–dihexoside or isomer	11.89	447(2), 285(100), 284(12), 151(4), 133(4)	609.1464	C_27_H_30_O_16_	[M−H]^−^	2.31	Flavonoid	[19]
92	Luteolin–pentoside–hexoside	12.03	285(77), 284(38), 151(4), 133(3)	579.1368	C_26_H_28_O_15_	[M−H]^−^	1.01	Flavonoid	[19,22]
93	Quercetagetin 7-methyl ether 3-neohesperidoside	12.09	639(86), 315(100), 314(24), 300(34), 299(29), 285(6), 271(40), 255(15), 243(20), 227(5)	639.1581	C_28_H_32_O_17_	[M−H]^−^	2.05	Flavonoid	[16,21]
94	Astilbin or isomer	12.13	303(14), 285(86), 179(14), 151(100), 125(20), 107(18)	449.1091	C_21_H_22_O_11_	[M−H]^−^	2.87	Flavonoid	[16,22]
95	Luteolin–hexoside	12.17	285(100), 284(39), 179(9), 151(65), 133(4)	447.0936	C_21_H_20_O_11_	[M−H]^−^	3.22	Flavonoid	[19,20]
96	Coumaric acid hexoside or isomer	12.52	163(100), 135(16), 117(7), 97(16)	325.0909	C_15_H_16_O_7_	[M+H]^+^	−2.53	Phenylpropanoids	[20]
97	Feruloyloctopamine or isomer	12.54	328(4), 311(19), 310(100), 161(89), 133(63)	328.1196	C_18_H_19_NO_5_	[M−H]^−^	1.53	Alkaloid	[20,21]
98	Luteolin–dexoyhexoside–hexoside	12.64	447(2), 285(63), 284(44), 151(3), 133(3)	593.1522	C_27_H_30_O_15_	[M−H]^−^	3.48	Flavonoid	[20]
99	Isoacteoside	12.73	461(9), 179(3), 161(100), 133(31), 113(12)	623.1995	C_29_H_36_O_15_	[M−H]^−^	2.21	Phenylpropanoids	[16,17,18,19,20]
100	Hydroxybenzoic acid	12.82	93(100)	137.0247	C_7_H_6_O_3_	[M−H]^−^	2.08	Organic acid	[16,17,18,19,20]
101	Solanigrine K	13.14	940(7), 899(43), 898(100), 753(19), 752(52), 163(10), 113(15), 101(19)	940.4922	C_47_H_75_NO_18_	[M−H]^−^	1.12	Alkaloid	[21]
102	Solanigrine D or isomer	13.14	754(100), 736(15), 718(19), 85(9)	754.4358	C_39_H_63_NO_13_	[M+H]^+^	−1.89	Alkaloid	[21]
103	Plantamajoside isomer	13.18	639(49), 447(27), 179(30), 161(100), 135(22), 133(34)	639.1942	C_29_H_36_O_16_	[M−H]^−^	1.81	Phenylethanoid glycoside	[20]
104	3, 4-dimethoxyphenyl-acrylamide	13.27	344(5), 177(100), 151(7), 145(41), 117(17)	344.1484	C_19_H_21_NO_5_	[M+H]^+^	−2.63	Alkaloid	[20]
105	Verbascoside	13.60	461(6), 179(2), 161(100), 133(30), 113(12)	623.1990	C_29_H_36_O_15_	[M−H]^−^	1.43	Phenylpropanoids	[16,17,18,19,20]
106	Kaempferol-3-O-rutinoside	13.76	285(68), 284(52), 255(43), 227(26), 151(4)	593.1523	C_27_H_30_O_15_	[M−H]^−^	3.79	Flavonoid	[19,22]
107	Astilbin or isomer	13.79	303(17), 285(51), 179(15), 151(100), 125(66), 107(19)	449.1099	C_21_H_22_O_11_	[M−H]^−^	3.57	Flavonoid	[16]
108	Isorhamnetin–dexoyhexoside–hexoside	14.18	623(100), 461(6), 161(65), 135(10), 133(23), 113(10)	623.1633	C_28_H_32_O_16_	[M−H]^−^	2.13	Flavonoid	[21]
109	4, 5-Dicaffeoylquinic acid	14.24	353(83), 191(100), 179(62), 135(69)	515.1204	C_25_H_24_O_12_	[M−H]^−^	1.67	Organic acid	[17,21]
110	Coumaric acid hexoside or isomer	14.26	163(100), 135(17), 117(10), 97(3)	325.0908	C_15_H_16_O_7_	[M+H]^+^	−2.81	Phenylpropanoids	[20]
111	Skimmin	14.50	325(100), 307(16), 163(100), 145(11), 135(19), 117(9)	325.0908	C_15_H_16_O_8_	[M+H]^+^	−3.1	Phenylpropanoids	[20]
112	Isosctoside or isomer	14.50	623(100), 461(8), 179(3), 161(80), 135(17), 133(23), 113(15)	623.1989	C_29_H_36_O_15_	[M−H]^−^	1.22	Organic acid	[16,20]
113	Kaempferol-3-O-glucoside	14.51	285(48), 284(69), 151(8)	447.0943	C_21_H_20_O_11_	[M−H]^−^	2.31	Flavonoid	[20,22]
114	Solanigrine D or isomer	14.57	754(100), 736(4), 430(9), 85(13)	754.4355	C_39_H_63_NO_13_	[M+H]^+^	−2.3	Alkaloid	[21]
115	Trifolin	14.65	447(100), 301(80), 300(91), 285(21), 271(49), 255(49), 243(11), 179(14), 151(48), 107(10)	447.0946	C_21_H_20_O_11_	[M−H^]−^	2.93	Flavonoid	[16,17,18,19,20]
116	Taxifolin or isomer	14.68	305(7), 287(11), 259(38), 231(32), 213(9), 153(100), 149(37), 123(33)	305.0647	C_15_H_12_O_7_	[M+H]^+^	−2.01	Flavonoid	[16]
117	Astilbin or isomer	14.68	449(14), 303(17), 285(52), 255(11), 179(17), 151(100), 107(19)	449.1093	C_21_H_22_O_11_	[M−H]^−^	0.86	Flavonoid	[16]
118	Quercetin–hexoside or isomer	15.11	301(100), 300(5), 273(2), 179(17), 151(39), 107(10)	463.0893	C_21_H_20_O_12_	[M−H]^−^	3.22	Flavonoid	[22]
119	Homoesperetin–dexoyhexoside–hexoside	15.16	623(75), 461(9), 179(3), 161(100), 135(14), 133(33), 113(14)	623.1991	C_29_H_36_O_15_	[M−H]^−^	1.53	Flavonoid	[16,17,18,19,20]
120	Luteolin–hexoside	15.20	285(100), 151(8), 133(8)	447.0941	C_21_H_20_O_11_	[M−H]^−^	4.24	Flavonoid	[16,19,20]
121	Quercetin–hexoside or isomer	15.26	463(5), 301(100), 300(5), 179(18), 151(48)	463.0892	C_21_H_20_O_12_	[M−H]^−^	1.77	Flavonoid	[16,17,18,19,20]
122	Solanigrine D or isomer	15.26	754(100), 574(25), 253(8)	754.4360	C_39_H_63_NO_13_	[M+H]^+^	−1.65	Alkaloid	[21]
123	Apigenin–hexoside	15.31	268(100), 151(10)	431.0991	C_21_H_20_O_10_	[M−H]^−^	2.73	Flavonoid	[19]
124	Isorhamnetin-3-O-glucoside	15.41	314(47), 285(23), 271(38), 243(43), 151(3)	477.1046	C_22_H_22_O_12_	[M−H]^−^	3.96	Flavonoid	[20,21,22]
125	Apigetrin	15.46	433(1), 271(100)	433.1118	C_21_H_20_O_10_	[M+H]^+^	−2.56	Flavonoid	[19,20]
126	Cynaroside or isomer	16.12	447(88), 285(40), 284(100), 151(45), 135(10)	447.0945	C_21_H_20_O_11_	[M−H]^−^	2.81	Flavonoid	[20,22]
127	Homoplantaginin	16.60	297(10), 283(54), 255(75)	461.1098	C_22_H_22_O_11_	[M−H]^−^	3.33	Flavonoid	[20]
128	Calceolarioside B	17.01	477(92), 337(28), 175(100), 161(84), 124(23), 123(31)	477.1410	C_23_H_26_O_11_	[M−H]^−^	1.64	Phenylethanoid glycoside	[19,22]
129	Physalin G	17.11	507(4), 497(11), 481(20), 463(18), 193(5)135(100)	525.1774	C_28_H_30_O_10_	[M−H]^−^	3.51	Steroids	[20]
130	Luteolin–hexoside or isomer	17.21	285(100), 151(7), 133(8)	447.0938	C_21_H_20_O_11_	[M−H]^−^	3.56	Flavonoid	[16,20]
131	Trilobatin	17.37	435(44), 420(100), 391(56), 389(57), 335(48), 272(29), 234(77), 206(47), 190(62), 179(39), 135(77)	435.1304	C_21_H_24_O_10_	[M−H]^−^	1.75	Flavonoid	[16]
132	Luteolin–hexoside or isomer	17.43	285(100), 284(5), 151(7), 133(8)	447.0941	C_21_H_20_O_11_	[M−H]^−^	1.41	Flavonoid	[16,20]
133	Isorhamnetin–hexoside or isomer	17.57	315(20), 314(55), 299(58), 285(8), 271(49), 151(5)	477.1042	C_16_H_12_O_7_	[M−H]^−^	3.01	Flavonoid	[20,21,22]
134	Quercetin–hexoside or isomer	17.63	463(41), 301(100), 300(5), 179(26), 151(52)	463.0888	C_21_H_20_O_12_	[M−H]^−^	0.95	Flavonoid	[16,20,22]
135	Solanigrine B or isomer	17.91	914(100), 897(6), 896(11), 85(13)	914.4714	C_45_H_71_NO_18_	[M+H]^+^	−3.3	Alkaloid	[21]
136	Naringenin–hexoside	18.09	271(100), 151(94), 119(24), 107(22)	433.1151	C_21_H_22_O_10_	[M−H]^−^	3.01	Flavonoid	[22]
137	Solasonine	18.43	866(11), 848(14), 114(4)	884.4984	C_45_H_73_NO_16_	[M+H]^+^	−2.04	Alkaloid	[21]
138	Favolon B	18.84	575(100), 213(11), 157(6)	575.3563	C_33_H_50_O_8_	[M+H]^+^	−2.65	Other	[21]
139	Genistin	19.00	268(59), 239(66), 211(68)	431.0988	C_21_H_20_O_10_	[M−H]^−^	3.43	Flavonoid	[20,22]
140	Apigenin 7-(6”-malonylglucoside)	19.22	519(3), 271(100)	519.1124	C_24_H_22_O_13_	[M+H]^+^	−1.84	Flavonoid	[20]
141	Eriodictyol	19.30	177(3), 161(2), 151(86), 135(100), 107(17)	287.0564	C_15_H_12_O_6_	[M−H]^−^	1.12	Flavonoid	[16,20,22]
142	Smilaxchinoside C	19.50	901(40), 597(16), 415(46), 271(33), 253(16), 157(13), 145(29)	901.4772	C_45_H_72_O_18_	[M+H]^+^	−2.19	Phenylpropanoids	[19]
143	Luteolin	20.10	241(2), 199(3), 175(37), 151(13), 133(37)	285.0408	C_15_H_10_O_6_	[M−H]^−^	4.13	Flavonoid	[16,20,22]
144	Solanine	20.21	868(100), 850(8), 85(10)	868.5028	C_45_H_73_NO_15_	[M+H]^+^	−2.87	Alkaloid	[21]
145	Quercetin	20.21	301(100), 273(5), 179(28), 151(89), 121(26), 107(28)	301.0341	C_15_H_10_O_7_	[M−H]^−^	−3.93	Flavonoid	[21,22]
146	Peimine	20.33	432(100), 414(11), 161(14)	432.3462	C_27_H_45_NO_3_	[M+H]^+^	−2.32	Alkaloid	[19]
147	Smilaside B	20.47	735(9), 675(7), 193(12), 175(100), 160(78), 132(21)	735.2159	C_34_H_40_O_18_	[M−H]^−^	2.3	Phenylpropanoids	[16]
148	Physalin J	20.58	497(14), 463(16), 341(35), 323(31), 149(25), 135(100), 121(13)	525.1769	C_28_H_30_O_10_	[M−H]^−^	2.69	Steroids	[19]
149	Schidigerasponin A3	20.59	901(12), 739(25), 415(24), 271(61), 253(100), 157(21)	901.4777	C_45_H_72_O_18_	[M+H]^+^	−1.64	Phenylpropanoids	[19]
150	Verruculin	20.60	917(100), 755(9), 101(17)	917.4761	C_47_H_66_N_8_O_11_	[M−H]^−^	−1.84	Alkaloid	[19]
151	Trigoneoside Xb	20.70	919(100), 757(11)	919.4923	C_45_H_76_O_19_	[M−H]^−^	1.56	Steroids	[16,19]
152	Colisporifungin	20.71	903(16), 741(80), 597(50), 255(100), 161(48)	903.4934	C_47_H_66_N_8_O_10_	[M+H]^+^	−4.56	Alkaloid	[19]
153	Timosaponin A1	20.71	579(27), 417(48), 273(100), 255(33), 161(55), 147(15)	579.3885	C_33_H_54_O_8_	[M+H]^+^	−1.13	Flavonoid	[19,21]
154	Quercetin–methyl	20.93	300(100), 271(60), 255(26), 243(13), 227(3)	315.0517	C_16_H_12_O_7_	[M−H]^−^	1.56	Flavonoid	[16,22]
155	Isorhamnetin or isomer	21.05	315(100), 300(77), 271(6), 255(3), 243(3), 227(3), 151(20)	317.0649	C_16_H_12_O_7_	[M+H]^+^	−2.01	Flavonoid	[22]
156	Khasianine	21.09	722(100), 704(9), 157(3), 85(10)	722.4457	C_39_H_63_NO_11_	[M+H]^+^	−2.28	Alkaloid	[21,22]
157	Physalin E	21.20	507(37), 497(31), 463(42), 193(18), 149(62), 135(100), 121(36)	525.1774	C_28_H_30_O_10_	[M−H]^−^	3.62	Steroids	[19]
158	Naringenin	21.47	151(70), 119(75), 107(26)	271.0615	C_15_H_12_O_5_	[M−H]^−^	1.1	Flavonoid	[16,17,18,19,20]
159	Silybin B	21.76	481(79), 453(25), 301(40), 179(29), 151(24), 125(100), 107(6)	481.1150	C_25_H_22_O_10_	[M−H]^−^	1.75	Flavonoid	[22]
160	Smilaside A	21.76	777(8), 735(3), 193(11), 175(100), 160(83), 134(14), 132(23)	777.2268	C_36_H_42_O_19_	[M−H]^−^	2.64	Phenylpropanoids	[16]
161	Apigenin	21.76	151(12), 117(23), 107(7)	269.0461	C_15_H_10_O_5_	[M−H]^−^	1.65	Flavonoid	[16,19]
162	Hesperetin	21.85	286(10), 196(6), 164(37), 151(47), 134(26), 107(18)	301.0724	C_16_H_14_O_6_	[M−H]^−^	−0.44	Flavonoid	[16,20,22]
163	Smilaside G	22.12	809(27), 633(28), 367(21), 175(71), 160(76), 145(100), 117(29)	809.2316	C_40_H_42_O_18_	[M−H]^−^	2.21	Phenylpropanoids	[16]
164	Silybin	22.29	481(63), 453(34), 301(7), 179(17), 151(15), 125(100)	481.1151	C_25_H_22_O_10_	[M−H]^−^	1.82	Flavonoid	[22]
165	Physalin A	22.31	507(10), 497(7), 463(6), 193(11), 149(100), 121(70)	525.1766	C_28_H_30_O_10_	[M−H]^−^	2.12	Steroids	[19]
166	Smilaside C	22.33	839(22), 663(26), 193(10), 175(91), 160(100), 145(73), 132(28), 117(27)	839.2422	C_41_H_44_O_19_	[M−H]^−^	2.12	Phenylpropanoids	[16]
167	Isorhamnetin or isomer	22.53	315(51), 300(100), 271(53), 255(24), 243(13), 227(3), 151(2)	317.0651	C_16_H_12_O_7_	[M+H]^+^	−1.62	Flavonoid	[22]
168	Physalin N or isomer	22.62	507(17), 479(14), 463(8), 193(6), 149(100), 121(68)	525.1766	C_28_H_30_O_10_	[M−H]^−^	2.12	Steroids	[19]
169	Diosmetin	22.68	284(100), 255(83), 227(59), 151(3)	299.0566	C_16_H_12_O_6_	[M−H]^−^	0.17	Flavonoid	[22]
170	Chrysomycin A	22.83	507(20), 445(13), 419(30), 401(12), 375(19), 357(16), 331(13), 173(100), 157(55)	507.1665	C_28_H_28_O_9_	[M−H]^−^	0.56	Other	[19]
171	Curtisian Q	22.83	525(52), 507(42), 497(44), 481(16), 479(27), 193(12), 173(11), 149(100)	561.1539	C_34_H_26_O_8_	[M−H]^−^	−2.88	Phenylethanoid glycoside	[19]
172	Homodimericin A	22.83	491(100), 419(7), 333(16), 197(11), 169(13), 155(74)	491.1695	C_28_H_26_O_8_	[M+H]^+^	−1.18	Other	[19]
173	Smilaside E	23.09	881(29), 821(7), 705(26), 175(100), 161(10), 160(92), 145(74), 132(29), 117(26)	881.2532	C_43_H_46_O_20_	[M−H]^−^	2.57	Phenylpropanoids	[16]
174	Corchorifatty acid F	23.22	327(33), 309(15), 201(24), 171(100), 137(27), 125(6)	327.2165	C_18_H_32_O_5_	[M−H]^−^	−3.58	Other	[19,21]
175	Aspermeroterpene B	23.36	527(19), 447(23), 403(23), 323(38), 173(15), 149(100)	527.1931	C_28_H_32_O_10_	[M−H]^−^	1.48	Other	[19]
176	Physalin N or isomer	23.52	507(8), 479(10), 463(9), 193(11), 149(100), 121(98)	525.1772	C_28_H_30_O_10_	[M−H]^−^	3.28	Steroids	[19]
177	Alldimycin C	23.71	526(6), 509(100), 491(7), 171(11)	544.2169	C_28_H_33_NO_10_	[M+H]^+^	−1.48	Alkaloid	[19]
178	Tetrahydroxy–dimethoxyflavone	23.88	330(67), 315(5), 287(3), 151(20)	345.0618	C_17_H_14_O_8_	[M−H]^−^	3.86	Flavonoid	[19]
179	Phylloflavanine	24.28	177(80), 147(100), 145(40), 117(17)	661.1902	C_35_H_32_O_13_	[M+H]^+^	−2.09	Flavonoid	[16]
180	Trihydroxy–octadecenoic acid	24.51	201(10), 171(56), 127(8)	329.2337	C_18_H_34_O_5_	[M−H]^−^	0.99	Other	[16,17,18,19,20]
181	Arjungenin	24.56	503(100), 401(3)	503.3389	C_30_H_48_O_6_	[M−H]^−^	2.21	Other	[17]
182	Chrysin	25.12	209(3), 143(5), 107(3)	253.0511	C_15_H_10_O_4_	[M−H]^−^	1.45	Flavonoid	[22]
183	Galangin	25.53	213(3), 169(3)	269.0461	C_15_H_10_O_5_	[M−H]^−^	1.87	Flavonoid	[22]
184	Licanic acid	26.38	275(100), 257(23), 229(19)	293.2121	C_18_H_28_O_3_	[M−H]^−^	3.42	Organic acid	[16,17,18,19,20]
185	Isoorientin 2″-O-(E)-p-coumarate	26.73	549(8), 209(58), 425(17), 121(100)	593.1314	C_30_H_26_O_13_	[M−H]^−^	2.26	Flavonoid	[16,17,18,19,20]
186	Kamlolenic acid	27.47	277(44), 259(3)	295.226	C_18_H_30_O_3_	[M+H]^+^	3.42	Organic acid	[22]
187	Ganoleucoin K	29.05	391(32), 279(26), 255(94), 197(100), 152(50), 107(9)	671.3070	C_36_H_48_O_12_	[M−H]^−^	−0.48	Other	[19]
188	Eleostearic acid	29.37	123(11), 109(22), 95(65), 81(87), 67(100)	279.2310	C_18_H_30_O_2_	[M+H]^+^	−2.77	Organic acid	[16,17,18,19,20]

**Table 2:** Prototype blood components and their metabolites of MZBZ.

No.	Transformations	tR/min	Parent Aglycone	Formula	*m*/*z*	Adduct Ion	Error/ppm	MS/MS
P1	/	4.17	Geniposidic acid	C_16_H_22_O_10_	373.1147	[M−H]^−^	1.87	211(34), 193(5), 167(24), 149(53), 123(100)
P2	/	4.66	Catechol	C_6_H_6_O_2_	109.0297	[M−H]^−^	1.71	91(3), 67(12)
M1	Glucuronide Conjugation	4.97	Taxifolin	C_21_H_20_O_13_	479.0843	[M−H]^−^	4.741	303(100), 285(79), 125(87)
M2	Glucuronide Conjugation	5.94	Taxifolin	C_21_H_20_O_13_	479.0841	[M−H]^−^	4.43	303(100), 285(8), 125(92)
P3	/	5.99	2,5-Dihydroxybenzoic acid	C_7_H_6_O_4_	153.0197	[M−H]^−^	2.48	109(100), 108(84)
P4	/	6.44	Neochlorogenic acid	C_16_H_18_O_9_	353.0882	[M−H]^−^	1.72	191(100), 179(4), 173(5), 135(8)
M3	Hydration, Glucuronide Conjugation	6.54	Apigenin	C_21_H_20_O_12_	463.0893	[M−H]^−^	4.79	287(100), 259(44), 243(10), 125(61)
P5	/	6.70	Cryptochlorogenic acid	C_16_H_18_O_9_	353.0885	[M−H]^−^	1.88	191(62), 179(68), 173(100), 135(78), 93(8), 85(12)
M4	Oxidation, Glucournid Conjugation	6.87	Hesperetin	C_22_H_22_O_13_	493.1000	[M−H]^−^	4.75	317(100), 289(35)
P6	/	7.17	Caffeic acid	C_9_H_8_O_4_	179.0354	[M−H]^−^	2.13	135(100)
M5	Hydration, Glucuronide Conjugation	7.23	Apigenin	C_21_H_20_O_12_	463.0894	[M−H]^−^	4.94	287(100), 259(39), 243(11), 125(59)
M6	Oxidation, Glucournid Conjugation	7.51	Hesperetin	C_22_H_22_O_13_	493.1000	[M−H]^−^	4.63	317(100), 289(33)
M7	Oxidation, Glucournid Conjugation	8.27	Hesperetin	C_22_H_22_O_13_	493.0998	[M−H]^−^	4.37	317(100), 289(41)
M8	Sulfation	9.04	Taxifolin	C_11_H_12_O_5_	383.0081	[M−H]^−^	−3.09	383(14), 339(100), 231(25)
M9	Sulfation	11.02	Isorhamnetin	C_16_H_12_O_10_S	395.0085	[M−H]^−^	4.4	315(100), 300(28), 151(28)
P8	/	12.04	Quercetin–hexoside	C_21_H_20_O_12_	463.0897	[M−H]^−^	3.28	301(59), 300(83), 271(49), 151(13)
P9	/	12.40	Astilbin or isomer	C_21_H_22_O_11_	449.1103	[M−H]^−^	2.98	303(18), 285(48), 179(15), 151(100), 125(20), 107(17)
P9	/	12.42	Baicalein 6-glucuronide	C_21_H_18_O_11_	445.0787	[M−H]^−^	2.40	269(100), 175(3), 113(15), 97(29)
P10	/	13.09	4-Hydroxybenzoic acid	C_7_H_6_O_3_	137.0247	[M−H]^−^	2.19	93(100)
P11	/	14.65	Homoesperetin 7-rutinoside	C_29_H_36_O_15_	623.1998	[M−H]^−^	2.60	580(10), 402(9), 161(100), 133(28)
P12	/	14.81	Astilbin or isomer	C_21_H_22_O_11_	449.1101	[M−H]^−^	2.70	303(21), 285(64), 179(21), 151(100), 125(27), 107(22)
M10	Glucournide Conjugation	16.39	isorhamnetin	C_22_H_20_O_13_	491.0844	[M−H]^−^	4.75	315(100), 300(73), 271(16), 151(8)
P13	/	16.60	Dihydrokaempferide 3-glucuronide	C_22_H_22_O_12_	477.1050	[M−H]^−^	2.30	301(100), 175(22), 151(44), 134(15), 113(47)
M11	Glucournide Conjugation	17.31	isorhamnetin	C_22_H_20_O_13_	491.0844	[M−H]^−^	4.75	315(94), 300(100), 271(4), 151(16)
M12	Glucournide Conjugation	18.85	isorhamnetin	C_22_H_20_O_13_	491.0840	[M−H]^−^	4.06	315(100), 300(92), 271(21), 151(20)
M13	Sulfation	19.55	Luteolin	C_15_H_10_O_9_S	364.9979	[M−H]^−^	1.61	285(100), 257(7), 229(2), 151(17), 133(5)
P14	/	19.61	Engeletin	C_21_H_22_O_10_	433.1151	[M−H]^−^	2.51	257(100), 242(4), 175(30), 113(71), 85(33)
M14	Sulfation	19.62	Quercetin	C_15_H_10_O_10_S	380.9931	[M−H]^−^	2.39	301(100), 179(20), 151(53), 107(14)
M15	Sulfation	19.78	Isorhamnetin	C_16_H_12_O_10_S	395.0087	[M−H]^−^	4.95	315(100), 300(25), 151(34)
M16	Glucournide Conjugation	20.09	isorhamnetin	C_22_H_20_O_13_	491.0844	[M−H]^−^	4.81	315(100), 300(12), 271(22)
M17	Sulfation	20.09	Isorhamnetin	C_16_H_12_O_10_S	395.0085	[M−H]^−^	4.55	315(100), 300(61), 151(17)
P15	/	20.35	Luteolin	C_15_H_10_O_6_	285.0411	[M−H]^−^	2.36	241(2), 199(4), 175(4), 151(8), 133(27)
P16	/	20.60	Baicalein 6-glucuronide	C_21_H_18_O_11_	445.0787	[M−H]^−^	2.40	269(100), 175(3), 113(15), 97(29)
P17	/	27.91	12,27-dihydroxy-solasodine	C_27_H_43_NO_4_	446.3251	[M+H]^+^	−3.03	271(3), 133(3), 119(9), 85(60)
P18	/	32.16	Ursolic acid	C_30_H_48_O_3_	455.3545	[M−H]^−^	3.09	407(2), 255(3), 219(1), 145(2)

M represents metabolites of blood obsorbed components of MZBZ; P represents prototype components of MZBZ in blood serum.

**Table 3:** Effects of MZBZ on changes in serum and tissue cytokine levels in CNBP rats.

Group	IL-6 (pg/mL)	IL-17 (pg/mL)	TNF-α (pg/mL)
Normal	33.40 ± 6.42	18.85 ± 5.77	98.72 ± 11.8
Model	102.0 ± 11.6 ***	82.23 ± 12.1 ***	248.5 ± 44.2 ***
MZBZ	77.71 ± 9.31 ^ΔΔ^	64.13 ± 9.89 ^Δ^	184.6 ± 18.7 ^ΔΔ^

Compared with the normal group: *** *p* < 0.001; compared with the model group, ^Δ^ *p* < 0.05, ^ΔΔ^ *p* < 0.01.

**Table 4:** Relative expression ratio of key target proteins.

Group	p-p65/p65	p-akt/akt	TGF-β1/Actin	COX2/Actin	Bcl2/Atin
Normal	0.23 ± 0.03	0.29 ± 0.06	1.02 ± 0.14	0.94 ± 0.05	0.93 ± 0.06
Model	1.17 ± 0.08 ***	1.33 ± 0.16 ***	6.28 ± 0.55 ***	5.28 ± 0.55 ***	0.25 ± 0.55 ***
MZBZ	0.47 ± 0.07 ^ΔΔΔ^	0.72 ± 0.06 ^ΔΔΔ^	2.10 ± 0.52 ^ΔΔΔ^	2.50 ± 0.29 ^ΔΔ^	0.63 ± 0.07 ^ΔΔ^

Compared with the normal group: *** *p* < 0.001; compared with the model group: ^ΔΔ^ *p* < 0.01, ^ΔΔΔ^ *p* < 0.001.

## Data Availability

The original contributions presented in this study are included in the article. Further inquiries can be directed to the corresponding author.

## Abbreviations

The following abbreviations are used in this manuscript:

AKT	RAC-alpha serine/threonine-protein kinase.
BCL2	B-cell lymphoma-2.
CP	Chronic prostatitis.
CNP	Chronic nonbacterial prostatitis.
COX2	Cyclooxygenase-2.
ED	Erectile dysfunction.
GNPS	Global natural products social molecular networking.
IL-6	Interleukin-6.
IL-17	Interleukin-17.
LC-MS	Liquid chromatography–mass spectrometry.
MZBZ	Maizibizi Wan.
MMP-9	Matrix metalloproteinase-9.
NFK-B	Nuclear factor kappa-light-chain-enhancer of activated B cells.
TNF-a	Tumar necrosis factor a.
TGF-β1	Transforming growth factor-beta 1.
TCM	Traditional Chinese medicine.
